# PsmiR159b-*PsMYB65* module functions in the resumption of bud growth after endodormancy by affecting the cell cycle in tree peony

**DOI:** 10.1093/hr/uhae052

**Published:** 2024-02-23

**Authors:** Tao Zhang, Xinyu Wang, Yanchao Yuan, Shoujie Zhu, Chunying Liu, Yuxi Zhang, Shupeng Gai

**Affiliations:** College of Life Sciences, Qingdao Agricultural University, Qingdao, 266109, China; University Key Laboratory of Plant Biotechnology in Shandong Province, Qingdao, 266109, China; Department of Ornamental Horticulture, College of Horticulture, China Agricultural University, Beijing 100193, China; College of Life Sciences, Qingdao Agricultural University, Qingdao, 266109, China; University Key Laboratory of Plant Biotechnology in Shandong Province, Qingdao, 266109, China; College of Life Sciences, Qingdao Agricultural University, Qingdao, 266109, China; University Key Laboratory of Plant Biotechnology in Shandong Province, Qingdao, 266109, China; College of Life Sciences, Qingdao Agricultural University, Qingdao, 266109, China; University Key Laboratory of Plant Biotechnology in Shandong Province, Qingdao, 266109, China; College of Life Sciences, Qingdao Agricultural University, Qingdao, 266109, China; University Key Laboratory of Plant Biotechnology in Shandong Province, Qingdao, 266109, China; College of Life Sciences, Qingdao Agricultural University, Qingdao, 266109, China; University Key Laboratory of Plant Biotechnology in Shandong Province, Qingdao, 266109, China; College of Life Sciences, Qingdao Agricultural University, Qingdao, 266109, China; University Key Laboratory of Plant Biotechnology in Shandong Province, Qingdao, 266109, China

## Abstract

Bud endodormancy in perennial plants is a sophisticated system that adapts to seasonal climatic changes. Growth-promoting signals such as low temperature and gibberellins (GAs) are crucial for facilitating budbreak following endodormancy release (EDR). However, the regulatory mechanisms underlying GA-mediated budbreak in tree peony (*Paeonia suffruticosa*) remain unclear. In tree peony, the expression of PsmiR159b among three differentially expressed miR159 members was inhibited with the prolonged chilling, and overexpression of *PsMIR159b* delayed budbreak, whereas silencing *PsmiR159b* promoted budbreak after dormancy. *PsMYB65*, a downstream transcription factor in the GA pathway, was induced by prolonged chilling and exogenous GA_3_ treatments*. PsMYB65* was identified as a target of PsmiR159b, and promoted budbreak in tree peony. RNA-seq of *PsMYB65*-slienced buds revealed significant enrichment in the GO terms regulation of ‘cell cycle’ and ‘DNA replication’ among differentially expressed genes. Yeast one-hybrid and electrophoretic mobility shift assays demonstrated that PsMYB65 directly bound to the promoter of the type-D cyclin gene *PsCYCD3;1*. Dual-luciferase reporter assay indicated that PsMYB65 positively regulate *PsCYCD3;1* expression, suggesting that miR159b-*PsMYB65* module contributes to budbreak by influencing the cell cycle. Our findings revealed that the PsmiR159b-*PsMYB65* module functioned in budbreak after dormancy by regulating cell proliferation, providing valuable insights into the endodormancy release regulation mechanism.

## Introduction

In perennial woody plants, bud endodormancy plays a critical role in ensuring survival under the adverse environmental conditions present during winter, such as low temperature (LT) and dehydration stress. Bud dormancy consists of the following stages: endodormancy establishment, maintenance, endodormancy release (EDR), and budbreak [1, 2]. Growth cessation and endodormancy are established before winter, whereas EDR and resumption of normal growth occur only after a period of chilling in winter. Photoperiod and temperature are two main environmental factors controlling bud endodormancy in woody plant [3, 4]. In hybrid aspen (*Populus tremula × tremuloides*), short-day (SD) exposure leads to a reduction in *PtFT2* transcription, halting growth, and initiating bud formation to protect the shoot apical meristems [5, 6]. Under long day conditions, PtFT2 interacts with PtFD-like 1 to ensure high transcriptional levels of *PtAP1-like* [7], which activates key D-type cyclin (*PtCYCD*) transcripts to promote growth [8].

Gibberellins (GAs) and abscisic acid (ABA) play vital roles in the regulation of bud dormancy [6, 9]. Endogenous ABA levels increase during the establishment of endodormancy [10], whereas they decrease and GA levels increase during chilling-induced EDR [11]. Short vegetative phase (SVP)-like (SVL) acts as a regulatory molecule of the phytohormone pathway to hinder EDR [12]. Prolonged LT exposure impairs *SVL* expression, increases GA levels, and facilitates budbreak [13, 14]. Several studies have demonstrated that GA contributes to EDR and budbreak [4, 14]. External application of GA for accelerating dormancy release and budbreak has been demonstrated in numerous woody plants [15, 16]. The DELLA protein family acts as a negative regulator of the GA signaling pathway [17], and the DELLA gene, *PsRGL1*, inhibits dormancy release in tree peony [18]. However, GA-regulated downstream pathways in budbreak are poorly described.

In addition, accumulating evidences suggest that microRNAs (miRNAs) play a role in endodormancy regulation [19–21]. PsmiR172 functions in tree peony EDR by repressing *EARLY BUD-BREAK 1* (*EBB1*), which is a positive regulator of plant EDR [13, 22]. Based on small RNA sequencing, it has been reported that miR159s are differentially expressed between dormancy induction and EDR [21, 23]. However, the precise regulatory mechanisms of miR159 during bud dormancy require further investigation. miR159 is conserved in major land plants and targets a class of R2R3 MYB transcriptional factor (TF) named gibberellin MYB (GAMYB) [24]. GAMYB, a component of GA signaling, was first identified in barley (*Hordeum vulgare*). The GAMYB-like family contains three members in Arabidopsis: AtMYB33, AtMYB65, and AtMYB101 [25]. Previous studies have shown that GA induces the transcription of *GAMYB*, with notable expression in flowers [25, 26]. AtMYB33/65 affects various biological processes, including anther tapetal and pollen development, seed germination, and flowering in response to GA [27].

**Figure 1:**
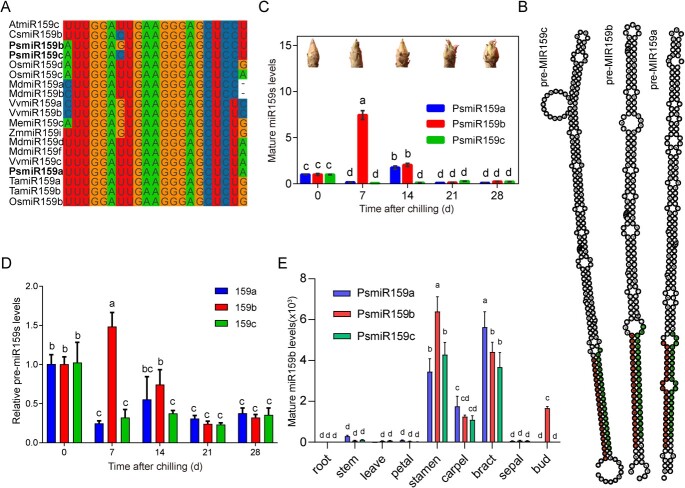
Identification and expression patterns of PsmiR159 family in tree peony ‘Luhehong’. (**A**) Alignment of mature miR159 sequences of tree peony and other known plants. Gene symbol with bold letters indicated the members of PsmiR159 family in tree peony. (**B**) Stem-loop structures of PsmiR159s. Red dots indicated the mature sequence of PsmiR159s. (**C**–**D**) Relative expression levels of the mature (**C**) and precursors (**D**) of PsmiR159s at 0, 7, 14, 21, 28 days of artificial chilling (DAC). Chilling perception phase was determined at 0 ~ 14 DAC, 14 ~ 21 DAC was the transition phase between endodormancy and endodormancy release (EDR), and 21 ~ 28 DAC was in ecodormancy state [29]. Pictures of tree peony buds were captured after being transferred to greenhouse for 10 days (18–22°C, 16/8 h light/dark). Data were represented as the mean ± standard deviation (SD) of three biological replicates. (**E**) The relative expression levels of mature miR159s in different tissues. Twenty-one-day chilling treated plants were transferred to greenhouse for 45 d, and the tissues were collected. The buds were a mix of 0, 7, 14, and 21 DAC. Data are shown as mean ± SD (*n* = 3) from three biological replicates (five buds in each replicate). Letters ‘a’ to ‘d’ in this figure indicate significant differences via one-way analysis of variance (ANOVA; Tukey test, *P* < 0.05).

Tree peony (*Paeonia suffruticosa* Andr.), an ancient woody ornamental plant in China, must experience a period of LT to break bud endodormancy. Previous study suggests that EDR is significantly triggered by prolonged chilling in tree peony. However, poor understanding about the molecular mechanisms of budbreak limits its industrial development [28]. Here, we found that a member of the miR159 family, PsmiR159b, and its target gene, *PsMYB65*, played important roles in tree peony budbreak. PsMYB65 directly bound to the promoter of *PsCYCD3;1* and activated its expression, which promotes cell proliferation, thereby facilitating budbreak in tree peony. These findings would provide new insights into the molecular mechanisms of growth resumption during dormancy transition and budbreak.

## Results

### Identification and expression analysis of PsmiR159 in tree peony

We previously identified some differentially expressed miRNAs during EDR, including three PsmiR159s (Fig. S1, see onlinesupplementary material), through small RNA sequencing. PsmiR159a, b, and c were identified in the tree peony genome. Here, we found that the lengths of mature PsmiR159a–c were highly conserved with 21 nucleotides (nt) (Fig. 1A). PsmiR159a–c shared the same 18 nt, differing only in the first, seventh, 20th, and 21st nucleotides at the 5′-ends (Fig. 1A). Then, the precursors of PsmiR159s were cloned, and the mature sequences were located in the 3′-stem arm of their stem–loop structures (Fig. 1B; File SS1, see online supplementary material).

**Figure 2:**
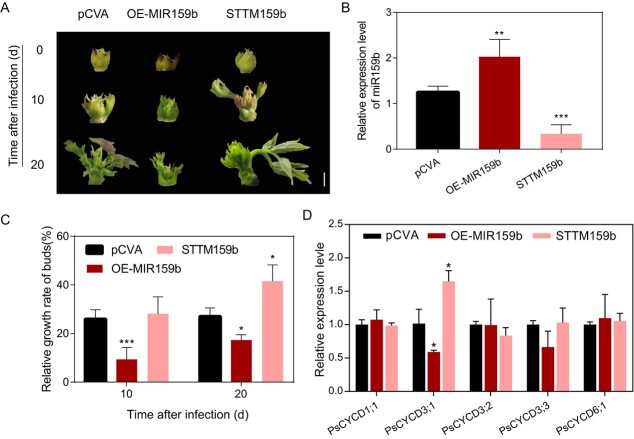
PsmiR159b inhibited tree peony budbreak. (**A**) Morphology of *PsMIR159b*-overexpressing (OE-*MIR159b*) and *PsMIR159b*-silenced (*STTM159b*) buds. pCVA, transgenic buds with empty pCVA vector. Buds were pictured at 10 and 20 d after infection (DAI). Scale bar, 5 mm. (**B**) Relative level of *PsmiR159b* at 10 DAI. Data were shown as mean ± SD (*n* = 3). (**C**) Relative growth rate of OE-*MIR159b* and *STTM159b* buds at 10 and 20 DAI. Data were shown as mean ± SD (*n* = 6). (**D**) Expression levels of D-type cyclin (*CYCDs*) in OE-*MIR159b*, *STTM159b* buds and control at 10 DAI. Data were shown as mean ± SD (*n* = 3). Asterisk (*) indicated statistically significant differences via two-tailed Student’s *t* test (^*^P < 0.05, ^**^P < 0.01, and ^***^P < 0.001).

The expression patterns of PsmiR159s in tree peony buds were analysed during chilling-induced EDR (0 ~ 28 days of artificial chilling, DAC) using quantitative reverse transcription-polymerase chain reaction (RT-qPCR). PsmiR159a was inhibited by short-term chilling treatment (7 DAC), and reached a peak after 14 DAC, and then decreased until 28 DAC. PsmiR159b was induced by chilling treated for 7 days, followed by a sharp decrease with the prolonged chilling. PsmiR159c exhibited low expression levels throughout all stages (Fig. 1C). To confirm these results, we also detected the expression patterns of their precursors during chilling-induced EDR. The results showed that the precursor of PsmiR159s were concomitant with mature PsmiR159s (Fig. 1C and D). Overall, the RT-qPCR data revealed expression patterns that were similar to those obtained by high-throughput sequencing. In term of spatial distribution, tissues other than the buds were isolated at the early stages of flowering, and the buds were mixed at 0, 7, 14, and 21 DAC. The results showed that all PsmiR159s were mainly expressed in flower organs, especially the stamen and bract, whereas only PsmiR159b showed higher expression levels in mixed buds (0–21 DAC) than others (Fig. 1E).

### PsmiR159b represses budbreak in tree peony

Similar to previous observations [29], over half of apical buds burst for 14 DAC plant, while almost all of the apical buds burst for the 21 DAC treatment, which indicated that 14 ~ 21 DAC is the transition from endodormancy to EDR in tree peony ‘Luhehong’. Because effective low-temperature accumulation is necessary for tree peony bud dormancy release, only PsmiR195b was relatively highly expressed in the bud, and its expression level was down-regulated with the extension of chilling accumulation (14 and 21 DAC), suggesting that it might play an important role during bud dormancy release. To further explore the function of PsmiR159b in budbreak, *PsMIR159b* was overexpressed in tree peony buds via virus-based miRNA expression system (Fig. 2A). Compared to the control, miR159b levels in *PsMIR159b*-overexpressing (OE-*MIR159b*) buds increased by approximately 1.7-fold (Fig. 2B). In contrast, OE-*MIR159b* buds delayed bud sprouting (Fig. 2A), and the relative growth rate of buds significantly decreased at 10 days after infection (DAI) (Fig. 2C). Using the short tandem target mimic (STTM) approach, *PsMIR159b*-silenced (*STTM159b*) buds were generated [30] (Fig. 2A; Fig. S2, see online supplementary material). In *STTM159b* buds, miR159b expression levels were approximately 73% lower than those in the control (Fig. 2B). *STTM159b* transgenic buds accelerated bud sprouting with a higher relative growth rate compared with the pCVA control, especially at 20 DAI (Fig. 2A and C). These results indicated that PsmiR159b inhibits budbreak in tree peony.

Bud endodormancy is accompanied by the reactivation of the cell cycle, with *CYCD* genes showing increased expression during EDR and budbreak in tree peony and poplar [8, 15, 28]. Gene family analysis of *CYCD* was performed using the CYCD HMM profile (Pfam: 00134) in tree peony, and five members of CYCD family were obtained (Fig. S3, see online supplementary material). The expression levels of *PsCYCDs* in *STTM159b* and *OE-MIR159b* buds were evaluated. Among them, the transcripts of *PsCYCD3;1* significantly increased in *STTM159b* transgenic buds and decreased in *OE-MIR159b* buds (Fig. 2D).

### *PsMYB65* is a target of PsmiR159b in tree peony

**Figure 3:**
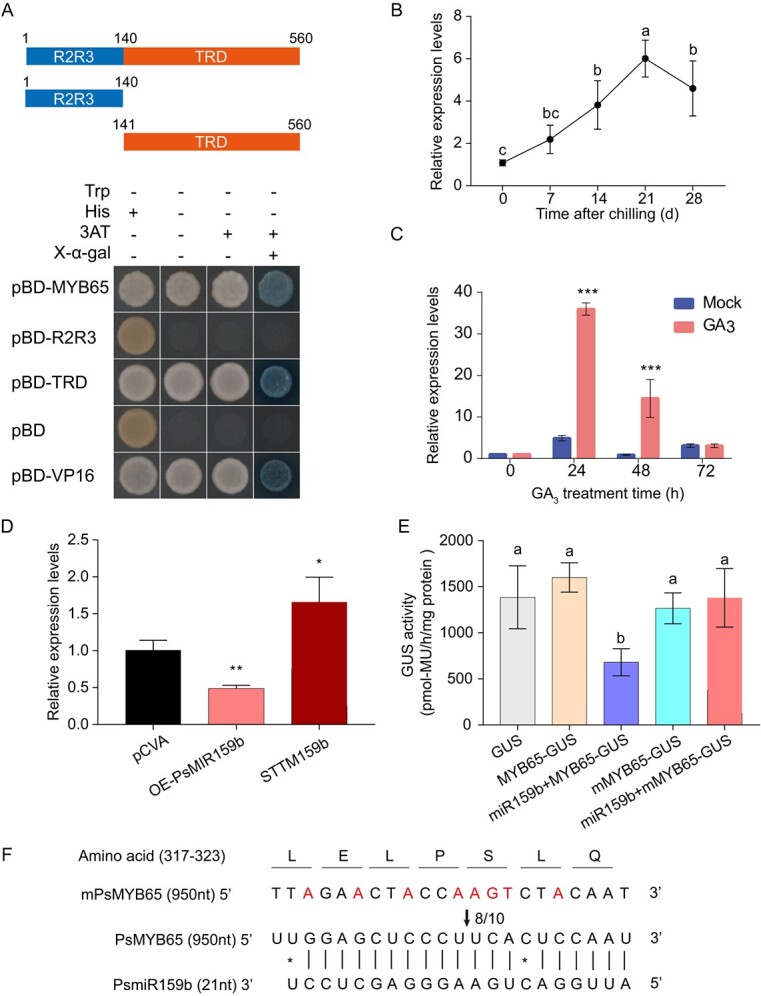
PsmiR159b targeted *PsMYB65* in tree peony. (**A**) The TRD domain was necessary for transcriptional activation activity of PsMYB65. R2R3, R2R3 domain. TRD, the putative transcription regulative domain. The transformants were selected on the SD/−Trp, SD/−Trp/-His, SD/−Trp/-His/ with 30 mM 3-amino-1,2,4-triazole (3-AT) and SD/−Trp/-His/X-α-Gal. The pBD-VP16 was as a positive control and pBD as a negative control. (**B**–**C**) Expression of *PsMYB65* during chilling-induced EDR (**B**), and after GA_3_ treatment (**C**). Data were represented as the mean ± SD (*n* = 5). Letters indicated the significant differences (one-way ANOVA, Duncan’s multiple range test). (**D**) Relative expression levels of *PsMYB65* in OE-*MIR159b* and *STTM159b* buds. pCVA, empty vector control. Values were represented as the mean ± SD of three biological replicates. Asterisk (*) indicated the significant differences via two-tailed Student’s *t*-test (^*^P < 0.05 and ^**^P < 0.01). (**E**) Enzyme activity of GUS in transformed tobacco leaves with *35S::GUS*, *35S::PsMYB65-GUS*, *35S::mPsMYB65-GUS*, *35S::PsMYB65-GUS* + *35S::PsMIR159b*, and *35S::mPsMYB65-GUS*+ *35S::PsMIR159b*. *mPsMYB65* indicated the synonymously mutated *PsMYB65* in the miR159b target region. Values were represented as the mean ± SD of six biological replicates, and letters over columns indicated the significant differences (one-way ANOVA, Duncan’s multiple range test). (**F**) Cleavage sites are identified via RNA ligase-mediated (RLM) 5′-rapid amplification of cDNA ends (RACE). A black arrow indicates the cleavage sites, and the numbers next to the arrow indicate the frequency of the cleavage fragments.

To identify the target gene of *PsmiR159b* in tree peony, we performed a target gene prediction analysis using PsRobot [31], and a *MYB* homolog with target site at 950–970 nt was obtained (Table S1, see online supplementary material). Phylogenetic analysis indicated that the predicted MYB was closely homologous to AtMYB65 (Figs S4 and S5A, see online supplementary material), and thus it was named PsMYB65. Alignment of amino acids showed that PsMYB65 contained an R2R3 domain and three conserved motifs (Fig. S5B, see online supplementary material). Subcellular localization analysis revealed that PsMYB65 was localized in the nucleus (Fig. S5C, see online supplementary material). Moreover, yeast assays demonstrated the transcriptional activation activity of PsMYB65, and its putative transcription regulatory domain (TRD) was essential for this activity (Fig. 3A).

Temporal–spatial expression patterns of *PsMYB65* were analysed using RT-qPCR. The transcripts of *PsMYB65* were upregulated during chilling-induced EDR and peaked at 21 DAC (Fig. 3B). *PsMYB65* also responded to GA_3_ treatment, with a significant increase after 24 h of treatment (Fig. 3C). Moreover, *PsMYB65* levels were higher in buds than other tissues (Fig. S5D, see online supplementary material).

The expression levels of *PsmiR159b* and *PsMYB65* exhibited an inverse pattern from 7 to 21 DAC (Figs 1D and 3 B). Additionally, we detected the expression levels of *PsMYB65* in OE-*MIR159b* and *STTM159b* buds. As expected, the transcripts of *PsMYB65* decreased by 52% in OE-*PsMIR159b* buds, while increased by approximately 1.6-flod in *STTM159b* transgenic buds (Fig. 3D). This led us to hypothesize that *PsMYB65* was a target of PsmiR159b during endodormancy transition. To verify this hypothesis, we generated the miR159b-insensitived *PsMYB65* (*mPsMYB65*) by synonymous mutation at the miR159 target site of PsMYB65. β-glucuronidase (GUS) reporter gene was used to construct a fusion protein with PsMYB65/mPsMYB65 under the control of the CaMV35S promoter, with *35S::GUS* as a control. Meanwhile, the precursor of PsmiR159b was driven by CaMV35S promoter (*35S::PsMIR159b*) to express PsmiR159b. Histochemical staining and GUS activity were used to evaluated GUS signals. The leaves transformed with *35S::GUS* and *35S::PsMYB65-GUS* exhibited strong GUS signals, whereas those co-expressing *35S::PsMYB65-GUS* and *35S::PsMIR159b* exhibited weaker GUS signals (Fig. 3E; Fig. S5E, see online supplementary material). Notably, GUS signals in leaves co-expressing *35S::mPsMYB65-GUS* and *35S::PsMIR159b* were not significantly different from those with only *35S::mPsMYB65-GUS* (Fig. 3E; Fig. S5E, see online supplementary material).

Next, *PsMYB65* mRNAs in buds at 7 DAC were used to examine the cleavage site by RLM-5′-RACE. The results showed that the cleavage site was located between the 10th and 11th bases from the 5′-end of the target site (Fig. 3F). Taken together, these results confirmed that *PsMYB65* was the target gene of PsmiR159b during EDR in tree peony.

### *PsMYB65* promotes budbreak in tree peony

**Figure 4:**
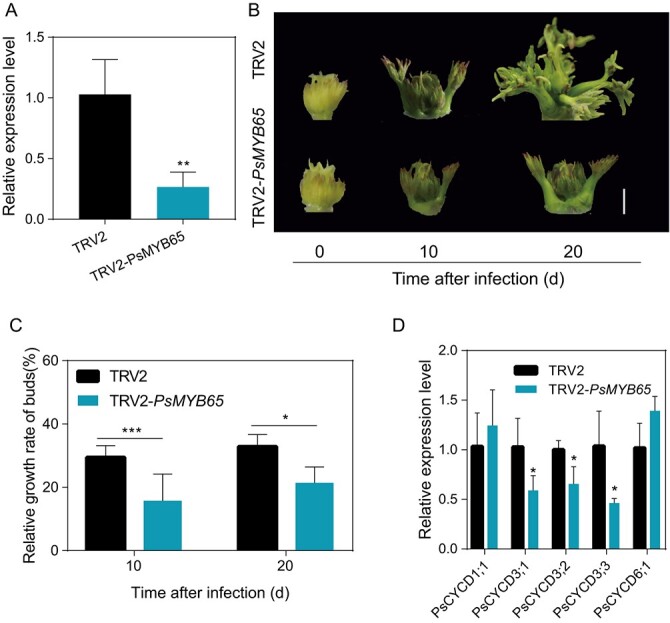
. *PsMYB65* accelerated budbreak in tree peony buds. (**A**) Expression levels of *PsMYB65* in TRV2-*PsMYB65* and control buds at 10 DAI. Data were represented as the mean ± SD (*n* = 5). (**B**) Morphology of TRV2-*PsMYB65* buds at 10 and 20 DAI. TRV2, pTRV2 empty vector control. TRV2-*PsMYB65*, *PsMYB65*-silenced buds. Scale bar, 5 mm. (**C**) Relative growth rate of TRV2-*PsMYB65* buds at 10 and 20 DAI. Data were represented as the mean ± SD (*n* = 5). (**D**) Expression levels of *PsCYCDs* in TRV2-*PsMYB65* and control buds at 10 DAI. Asterisks (*) in this figure indicated the statistically significant differences via two-tailed Student’s *t*-test (^*^P < 0.05, ^**^P < 0.01, and ^***^P < 0.001).

To further investigate the role of *PsMYB65* in bud endodormancy and/or budbreak regulation, *PsMYB65* was silenced via VIGS in tree peony buds. Expression levels of *PsMYB65* were determined at 10 DAI, and the average expression levels of *PsMYB65* decrease by 56% in TRV2-*PsMYB65* buds (Fig. 4A). Compared to TRV2 buds, TRV2-*PsMYB65* buds delayed sprouting (Fig. 4B), and the relative growth rate decreased by 48% after 10 DAI (Fig. 4C). As markers of the EDR state, five *PsCYCDs* transcripts were examined using RT-qPCR. We found that *PsCYCD3s* levels significantly decreased in *PsMYB65*-silenced buds (Fig. 4D).

### PsMYB65 affects cell cycle and cell division in tree peony buds

**Figure 5:**
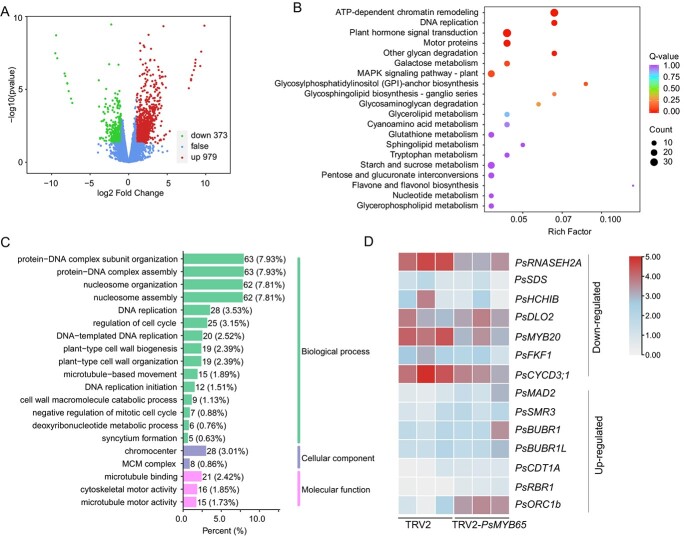
PsMYB65 affected cell cycle and cell division. (**A**) A volcano map of differentially expressed genes (DEGs) in RNA-seq of TRV2-*PsMYB65* and control buds. (**B**) Kyoto Encyclopedia of Genes and Genomes (KEGG) enrichment analysis of DEGs. (**C**) The top 20 GO terms of DEGs. (**D**) A heatmap of DEGs enriched into the term of cell cycle. DLO2, Downy mildew resistant 6 (DMR6)-LIKE OXYGENASE 2. FKF, flavin-binding, kelch repeat, F box. RNASEH2A, Ribonuclease H2 subunit A. SDS, SOLO DANCERS. HCHIB, basic chitinase. MAD2, Mitotic arrest-deficient 2. SMR3, Cyclin-dependent protein kinase inhibitor. BUBR1, BUB1-related 1. BUBR1L, BUBR1-LIKE. CDT1A, Arabidopsis homolog of yeast CDT1. RBR1, Retinoblastoma-related 1. ORC1b, Origin of replication complex subunit 1B.

To identify the downstream genes of *PsMYB65*, we performed RNA-seq of TRV2-*PsMYB65* buds, using TRV2 buds as a control (Table S2, see online supplementary material). In total, 1352 differentially expressed genes (DEGs) were obtained including 373 downregulated and 979 upregulated genes (Fig. 5A). Kyoto Encyclopedia of Genes and Genomes (KEGG) enrichment analysis revealed that these DEGs were enriched in several pathways, including ATP-dependent chromatin remodeling, DNA replication, and plant hormone signal transduction (Fig. 5B). Moreover, we performed Gene Ontology (GO) enrichment analysis to obtain further insight into the functions of these DEGs during budbreak. Among the top 20 GO terms, at least 10 GO terms were related to cell cycle and cell division (Fig. 5C). Moreover, the heatmap indicated that the expression levels of several known genes participated in cell cycle were down-regulated in *TRV2-PsMYB65* buds, such as *Ribonuclease H2 subunit A* (*RNASEH2A*) and *PsCYCD3;1*, whereas serval genes known to negatively regulate cell cycle were upregulated in *TRV2-PsMYB65* buds, such as *Mitotic arrest-deficient 2* (*MAD2*) and *Cyclin-dependent protein kinase inhibitor* (*SMR3*) (Fig. 5D). These findings suggested a potential connection between PsMYB65 and cell cycle regulation.

### PsMYB65 directly activates the expressions of *PsCYCD3;1*

**Figure 6:**
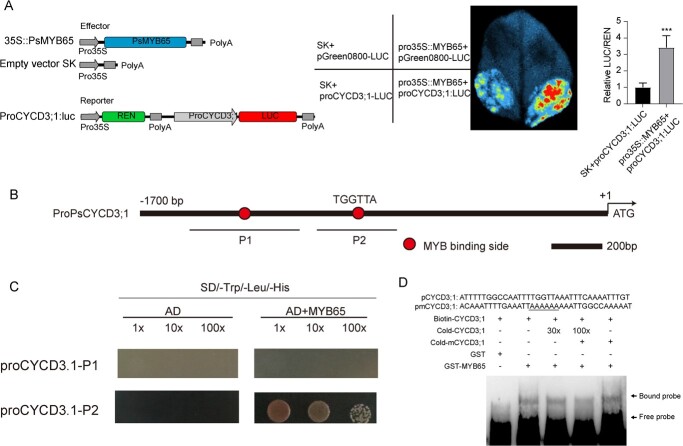
PsMYB65 directly bound to the promoter of *PsCYCD3;1* and activated its expression. (**A**) PsMYB65 activated the expression level of *PsCYCD3;1* using a dual-LUC reporter system. A schematic representation of the constructed dual LUC reporter system is shown on the left. Statistics are shown on the right-hand side. Data were represented as the mean ± SD (*n* = 8). Asterisks (*) indicated the significant differences between *ProPsCYCD3;1::LUC* + *Pro35S::PsMYB65* and empty vector (SK) + *ProPsCYCD3;1::LUC* via two-tailed Student’s *t*-test (^**^P < 0.01 and ^***^P < 0.001). (**B**) Analysis of MYB-binding sites in the promoter *PsCYCD3;1*. *PsCYCD3;1* promoter was truncated into two parts according to the distribution of potential MYB-binding sites (TGGTTA). (**C**) Y1H assay for the binding of PsMYB65 to *PsCYCD3;1* promoter. Fragments (P1 and P2 in B) of PsCYCD3;1 promoter were inserted into the pHIS2 vector. Co-expressing yeast cells were screened on SD/−Trp/−Leu/-His with 30 mM 3-AT. (**D**) EMSA assay of PsMYB65 binding to the TGGTTA motif in *PsCYCD3;1* promoter. pCYCD3;1 indicated the *PsCYCD3;1* promoter (−1139 to −1175 bp). pmCYCD3;1 indicated the mutation probes, and the mutation sites are underlined.

As known, EDR and budbreak accompanied by reactivation of cell cycle, and *CYCD* plays a critical role in EDR and budbreak [13, 28]. Among down-regulated DEGs enriched in cell cycle term, *PsCYCD3;1* was hindered in TRV2-*PsMYB65* buds but increased in *STTM159b* buds. Moreover, *PsCYCD3;1* was significantly induced by GA_3_ treatment (Fig. S6, see online supplementary material). Therefore, we explored whether PsMYB65 regulated the expression of *PsCYCD3;1*. Firstly, a 1700 bp promoter fragment of *PsCYCD3;1* was isolated and analysed using PlantCARE tool [32]. The analysis revealed two putative MYB-binding sites in *PsCYCD3;1* promoter (Fig. 5B), suggesting that PsMYB65 might regulate *PsCYCD3;1*. Furthermore, a dual-luciferase (LUC) reporter assay was performed to verify the relationship between PsMYB65 and *PsCYCD3;1*. LUC reporter gene, under the control of the promoter of *PsCYCD3;1* (*proPsCYCD3;1::LUC*), served as the reporter. *pro35S::PsMYB65* was used as the effector and empty vector SK as the control. The relative LUC activity in samples co-transformed *proPsCYCD3;1::LUC* and *pro35S::PsMYB65* was 3.5-fold higher than that in samples co-transformed with *proPsCYCD3;1:LUC* and empty vector SK (Fig. 6A).

Then, to confirm whether PsMYB65 directly bound to the promoter of *PsCYCD3;1*, a yeast one-hybrid (Y1H) assay was employed. The promoter of *PsCYCD3;1* was truncated into two parts based on the distribution of MYB-binding sites ‘TGGTTA’ (Fig. 6B). As shown in Fig. 6C, yeast cells harboring proPsCYCD3;1-P2 and AD-PsMYB65 could grow on synthetic defined (SD)-Trp-Leu-His medium with 30 mM of 3-amino-1,2,4-triazole (3-AT), whereas yeast cells with proPsCYCD3;1-P1/pHIS2 empty vector and AD-PsMYB65 could not grow. To further test the direct binding of PsMYB65 to the *PsCYCD3;1* promoter *in vitro*, we synthetized a probe containing this binding site, and labeled with biotin. Electrophoretic mobility shift assays (EMSAs) showed that the binding signal was observed when mixing recombinant PsMYB65-glutathione S-transferase (GST) and the labeled probe. The binding signal was enlarged in a dosage-dependent manner, whereas the binding signal was diminished with the addition of a competitive probe (Fig. 6D). These findings demonstrated that PsMYB65 directly bound to the promoter of *PsCYCD3;1* to regulate its expression.

### PsmiR159b–*PsMYB65* regulates cell division during budbreak

**Figure 7:**
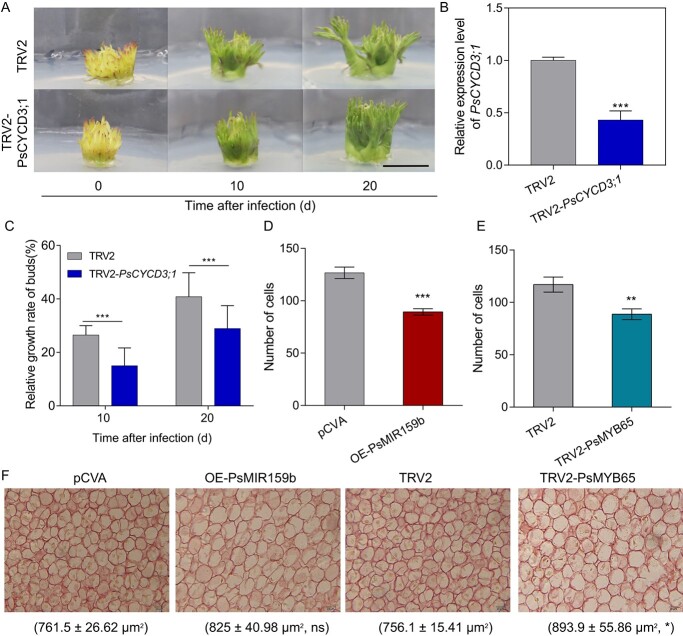
PsmiR159b–*PsMYB65* module affected cell cycle and cell division during budbreak. (**A**) Morphology of *PsCYCD3;1*-silenced buds at 10 and 20 DAI. TRV2, pTRV2 empty vector control. TRV2-*PsCYCD3;1*, *PsCYCD3;1*-silenced buds. OE-*PsMIR159b*, *PsMIR159b*-overexpressed buds. Scale bar, 5 mm. (**B**) Expression levels of *PsCYCD3;1* in TRV2-*PsCYCD3;1* and control buds. Data were represented as the mean ± SD (*n* = 3). (**C**) Relative growth rate of TRV2-*PsCYCD3;1* bud. Data were represented as the mean ± SD (*n* = 5). (**D**, **E**) Cell number of OE-*MIR159b* (**D**) and TRV2-*PsMYB65* buds (**E**). Data were represented as the mean of three different visual field of three buds with SD error bars. (**F**) Images of cell size in OE-*MIR159b* (**D**) and TRV2-*PsMYB65* buds (**E**). Cell area is given below the image (*n* = 5). Asterisks (*) in this figure indicate the statistically significant differences via two-tailed Student’s *t*-test (^*^P < 0.05, ^**^P < 0.01, and ^***^P < 0.001).

Considering that *PsMYB65* was the target of PsmiR159b, and PsMYB65 directly activated *PsCYCD3;1* expression (Figs 3 and 5), then we investigated the function of *PsCYCD3;1* during bud EDR and budbreak by VIGS (Fig. 7A). When compared with the control, the expression level of *PsCYCD3;1* was reduced by 57% in TRV2-*PsCYCD3;1* buds, and *PsCYCD3;1-*silenced buds exhibited slower sprouting with a lower relative growth rate after 10 and 20 DAI (Fig. 7C).

It is well known that CYCD play an important role in the cell cycle. Therefore, we assumed that PsmiR159b-PsMYB65 affected cell division during EDR and budbreak in tree peony buds. To confirm this hypothesis, we observed and quantified the cell number and size in OE*-PsMIR159b* and TRV2-*PsMYB65* buds by paraffin sections. The number of cells significantly decreased in OE*-PsMIR159b* and TRV2-*PsMYB65* buds (Fig. 7D and E), whereas the average cell sizes were bigger than the control buds (Fig. 7F). The reason might be that the overexpression of *PsMIR159b* and silencing of *PsMYB65* inhibited cell divisions. Overall, these results indicated that PsmiR159b–*PsMYB65* could modulate cell division, finally promoting budbreak in tree peony.

## Discussion

### miR159b targets PsMYB65 to regulate budbreak

miR159–GAMYBs play important roles in plant growth, flower organ development, flowering, fruit set, and defense response [25, 33–36]. However, their roles in EDR and budbreak remain unknown. Here, we reported for the first time that a miR159–*MYB65* module was involved in budbreak in tree peony by influencing the cell cycle. Genetic analyses revealed that *PsmiR159b* acted as a repressor in budbreak (Fig. 2). GUS signal and RLM-5′-RACE results demonstrated that *PsmiR159b* targeted *PsMYB65* (Fig. 3), a GAMYB gene, which promoted budbreak (Fig. 4). Furthermore, PsMYB65 was found to directly bind to the TGGTTA motif in *PsCYCD3;1* promoter to activate its expression (Fig. 5). These findings indicated that miR159b-*PsMYB65* module functioned in budbreak in tree peony.

To screen for EDR- or budbreak-associated genes, transcriptional profiling comparisons between the prolonged chilling and early chilling stages, other than no chilling, are recommended [28, 37]. In this study, PsmiR159b peaked at 7 DAC, but was significantly downregulated by prolonged chilling (Fig. 1), suggesting that PsmiR159b is a candidate EDR-associated gene. Subsequent results verified that PsmiR159b acts as a potential cell cycle inhibitor in the budbreak of tree peony. Interestingly, PsmiR159b was also low in abundance at 0 DAC (Fig. 1), indicating a dormant state. It is well known that the perception of SD is the main trigger for the establishment of bud dormancy, with low temperatures also playing a significant role in this process [4]. For PsmiR159b, SDs alone may not be sufficient to increase expression to levels that arrest cell division. MiRNAs usually target multiple transcripts and regulate several biological processes [38]. We speculated that there might be other unknown genes targeted by PsmiR159b in the dormancy processes in tree peony, and that the low expression of PsmiR159b was required to work well in the system. Another explanation is that factors other than PsmiR159b hinder cell division. In poplar, a reduction in FT2 expression plays a central role in bud formation and dormancy induction [5], probably caused by repressing *CYCD* expression [39]. Thus, the regulation and function of PsmiR159b during the entire dormancy process require further investigation.

In addition, PsmiR159s have been shown to have high expression levels in some floral organs such as stamens, bracts, and carpels during the early flowering stage (Fig. 1D), suggesting their potential role in the development of these organs. The involvement of miR159s in anther development has been reported in Arabidopsis, grapes, and *Brassica campestris* [33, 40, 41]. Thus, high expression of PsmiR159 in stamens may further influence anther development. Currently, there are not many reports on the relationship of miR159 with sepals and carpels. Based on the public database (TAIR), we found that AtmiR159b exhibited high expression levels in the sepals and carpels of Arabidopsis (Fig. S7, see online supplementary material). We speculated that the higher expression of miR159s in sepals and carpels was unrelated to the MYB65-CYCD pathway because flower buds still require cell growth and expansion to full bloom. Other targets of miR159b or biological processes may be involved in the early flowering stage, such as the miR159-CKX6 module, which regulates petal size through cytokinin catabolism in *Rosa hybrida* [42].

### PsMYB65 participates in GA-mediated budbreak

GAs are growth-promoting hormones in plants that play a major role in EDR and budbreak [4, 11, 14]. Activation of GA signaling is essential during EDR and budbreak in perennial woody plants [4]. Endogenous active GA levels increase from the dormant state to EDR stage in poplar and tree peony [3, 11]. The key genes involved in active GA biosynthesis, including *GA20ox* and *GA3ox*, are significantly up-regulated during EDR [28]. However, the mechanism by which the GA pathway influences the EDR remains unclear. Here, we identified that PsMYB65, a downstream GAMYB TF of GA signal transduction, was upregulated during EDR in tree peony (Fig. 3). Recent study has also reported that the expression of GAMYB was upregulated during EDR in *Culinary rhubarb* [43]. Our genetics and molecular biology findings indicated that *PsMYB65* positively regulated EDR and budbreak (Fig. 3). PsMYB65 promoted cell proliferation during EDR and budbreak by directly activating *PsCYCD3;1* expression (Fig. 5). Therefore, GA-regulated EDR and budbreak might be partially mediated by GAMYB.

### PsmiR159b-*PsMYB65* module regulates budbreak by affecting cell cycle

The cell cycle is fundamental to eukaryotic growth and also organ development and comprises a DNA synthesis phase (S), a mitotic phase (M), and two gap phases (G1 and G2). Each stage is tightly controlled by cyclin-dependent kinases (CDKs) and cyclin complexes [44]. Plant CDK proteins are divided into eight subgroups, of which A-type CDK (CDKA) and the plant-specific B-type CDK (CDKB) play a major role in cell cycle regulation [45]. type-A (CYCA), type-B (CYCB), and type-D (CYCD) are also crucial for controlling the cell cycle [44]. GAs have been shown to promote the expression of several CDKs and cyclins (*CDKA;2*, *CYCB2;1*, *CYCB2;2*, and *CDKD;1*), thus accelerating cell cycle progression [44], GAs application reduces the expression of the CDK inhibitors (CKIs) [46]. Xue *et al.* [47] finds that GAMYBs accelerate the cell cycle via the miR159 pathway in *Arabidopsis*. Although *CYCD* has been identified to play a positive role in growth of dormant buds [8], the transcriptional regulatory mechanisms of *CYCD* via GA signaling during budbreak after EDR still remain unclear. A few numbers of TFs have been found to regulate the expressions of *CYCDs* in EDR and budbreak, including *AIL1 (AINTEGUMENTALIKE 1)* and *EBB3 (EARLY BUD-BREAK 3)* [8, 13]. In this work, we identified PsMYB65 as a novel regulator of *CYCD,* directly promoting *PsCYCD3;1* expression during budbreak after dormancy (Fig. 5). Overexpression of *CYCD3;1* promotes cell proliferation and growth in poplar [48]. In tree peony, cell cycle was slowed down in *OE-PsMIR159b* and *PsMYB65*-slienced buds, suggested that miR159b–*PsMYB65* module regulated cell replication in budbreak after dormancy.

**Figure 8:**
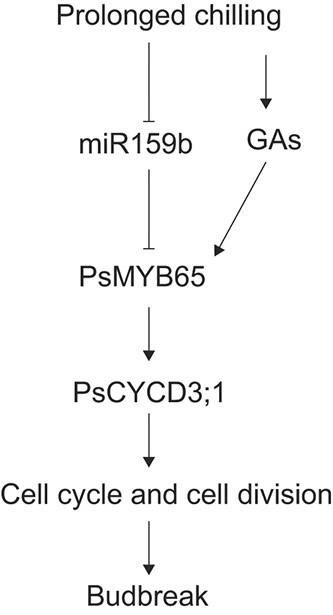
PsmiR159b–*PsMYB65* module regulates cell cycle during budbreak. A model of the PsmiR159b–*PsMYB65* module regulates budbreak in tree peony. After a prolonged chilling treatment, PsmiR159b level decreases, and hence, it could not sufficiently block *PsMYB65*, while the increasing endogenous GA promotes the expression of *PsMYB65*. Accumulated PsMYB65 accelerates cell cycle by promoting the expression of *PsCYCD3;1*, thereby accelerating bud burst.

In conclusion, we proposed a working model of PsmiR159b-*PsMYB65* regulating budbreak in tree peony (Fig. 8). Short-term chilling induced *PsmiR159b*, which targeted to *PsMYB65* and suppressed its transcripts, resulting in blockage of the downstream signaling. With the extension of chilling accumulation (14 and 21 DAC), the transcript of *PsmiR159b* decreased, and the expression of *PsMYB65* was up-regulated along with the increase of endogenous GAs. High levels of *PsMYB65* enhanced cell replication by directly activating *PsCYCD3;1* expression, and finally accelerated budbreak. Our findings provided novel insights into the miR159 function and GA signaling pathway in the regulation of tree peony budbreak after dormancy.

## Materials and methods

### Plant materials, growth conditions, and treatment

Four-year-old tree peony (*P. suffruticosa* cv. ‘Luhehong’) plants from Qingdao Agricultural University (Qingdao, China) were potted in late October 2018, which were moved to a refrigerator for artificial chilling treatment (0–4°C, 24 h/dark) as described by Zhang *et al.* [22]. Apical buds of uniform size were picked at 0, 7, 14, 21, and 28 days of artificial chilling (DAC), immediately frozen in liquid nitrogen, and stored at −80°C until use.

Plants after 7 DAC were transferred into a greenhouse (18–22°C, 16/8 h light/dark). The buds were then treated with 500 mg·L^−1^ gibberellic acid (48 880, Merck, Germany). Three plants were used for biological duplication, and at least three biological replicates were set.

To evaluate dormancy status, an equal number of unsampled plants after different DAC were transferred to a greenhouse, as previously described [49].

### Sequence processing and phylogenetic analysis

The sequences of *PsMYB65* and *PsCYCDs* were obtained from the tree peony transcriptome database [15], and their homologs in *Arabidopsis thaliana* were downloaded from the Arabidopsis Information Resource (https://www.arabidopsis.org/). Sequences were aligned using ClusterX (http://www.cluster-x.org/). Phylogenetic trees were constructed using MEGA 7.0 with the neighbor-joining method [50]. The stem–loop structures of *PsMIR159s* were elucidated using TBtools [51]. A schematic representation of PsMYB65 was drawn using IBS1.0.2 [52].

### Gene expression analysis

A total of 100 mg buds after different treatments including chilling and GA_3_ were used to extract total RNA using the MiniBEST Plant RNA Extraction Kit (TaKaRa, Dalian, China), and genomic DNA was removed using DNase I. Total RNA (1 μg) was used for cDNA synthesis by HiScript III RT SuperMix for qPCR (+gDNA wiper) (Vazyme, Nanjing, China). Quantitative reverse transcription-polymerase chain reaction (RT-qPCR) was performed to determine the relative expression levels using ChamQ Universal SYBR qPCR Master Mix (Vazyme, Nanjing, China). *PsActin* was used as an internal control [22].

For miRNA analysis, HiScript III 1st Strand cDNA Synthesis Kit was used for cDNA synthesis with a reaction solution containing PsmiR159bF/stem–loop universal-R primers, 1 μg RNA, RT Mix, and HiScript III Enzyme Mix. The mixture was incubated at 25°C for 5 min, 37°C for 45 min, and 85°C for 5 s. Then, SYBR RT-qPCR Kits (TakaRa, Dalian, China) were used to determine the miRNA expression levels. RT-qPCR was performed at 95°C for 1 min, followed by 40 cycles at 95°C for 30 s and 60°C for 30 s. *U6* was used as an internal control [21].

Relative expression levels of all genes and miRNAs were calculated using the 2^-ΔΔCT^ method [53]. All primers used for RT-qPCR are listed in Table S3 (see online supplementary material).

### Transient transformation in *Nicotiana benthamiana* leaves

To investigate the subcellular localization of PsMYB65, its coding sequences (CDS) without a termination codon were cloned into pSuper1300 harboring a green fluorescent protein (GFP) to generate the PsMYB65-GFP fusion vector. Nuclei were stained with 4′,6-diamidino-2-phenylindole (DAPI). All primers used for subcellular localization are listed in Table S3 (see online supplementary material).

To generate miR159b-insensitived PsMYB65, the target sequence of *PsmiR159b* in *PsMYB65*, GGAGCTCCCTTCACTC (951–966 bp), was synonymously mutated to AGAACTACCAAGTCTA via overlapping PCR. Mutated *PsMYB65* was named *mPsMYB65*. All primers used for *PsMYB65* mutation are listed in Table S3 (see online supplementary material).

To determine whether PsmiR159b targeted *PsMYB65*, a 182 bp *PsmiR159b* stem–loop sequence (File SS1, see onlinesupplementary material), the CDS sequences of *PsMYB65* and *mPsMYB65* were ligated into the pBI121 vector (harboring a GUS tag) to generate overexpression (OE) vectors *35S::PsmiR159b*, *35S::PsMYB65-GUS*, and *35S::mPsMYB65-GUS*. All primers used for vector construction are listed in Table S3 (see online supplementary material).

These constructed vectors were then transformed into *Agrobacterium tumefaciens* strain GV3101, which was further used to transform *N. benthamiana* leaves. Tobacco transformation was performed as previously described [22]. After infection for 3 d, GFP and DAPI fluorescence were observed via confocal microscopy (Leica SP8; Germany) at 488 and 360 nm, respectively. GUS staining and GUS protein activity analysis were performed using the method described by Jefferson *et al.* [54].

### RLM-5′-RACE

To determine the cleavage site of *PsMYB65* transcripts by PsmiR159b, an RNA ligase-mediated 5′-rapid amplification of cDNA ends (RLM-5′-RACE) assay was performed using a FirstChoice RLM-RACE kit (Invitrogen, San Diego, CA, USA). First, total RNA extracted from buds of 7 DAC was ligated to the 5′-adapter using T_4_ RNA ligase. cDNA was then synthesized using M-MLV reverse transcriptase at 42°C for 1 h. PCR product was cloned into the pMD18-T vector. Ten randomly selected recombinant single colonies were sequenced. All primers used in this assay are listed in Table S3 (see online supplementary material).

### Transformation of tree peony buds

A virus-based miRNA expression system was used for overexpressing of PsmiR159b as previously described with some modifications [22]. Dormant buds exposed to 7 DAC were used and hardly burst *in vivo* under growth conditions. Briefly, 182 bp *PsMIR159b* precursor (File SS1, see online supplementarymaterial) was ligated into pCVA vector to generate *PsMIR159b*-overexpressed vector (OE-*PsMIR159b*). For *PsMIR159b* silencing, the short tandem target mimic (STTM) of PsmiR159b (STTM159b; Figure S2, see online supplementary material) was constructed and then ligated into pCVA vector. Then, these recombinant vectors were transformed into *A. tumefaciens* strain EHA105.

For *PsMYB65* and *PsCYCD* silencing, VIGS was performed as previously described, with minor modifications [22]. Briefly, a specific 450 bp fragment of *PsMYB65* cDNA and a 367 bp fragment of *PsCYCD3;1* was introduced into pTRV2 vector (pTRV2*-PsMYB65* and pTRV2*-CYCD3;1*), respectively. Then, they were transformed into *A. tumefaciens* strain EHA105.

*A. tumefaciens* strains harboring the recombinant plasmids (OE-*PsMIR159b*, STTM159b, *TRV-PsCYCD3;1*, and TRV2-*PsMYB65*), empty plasmids (pCVA and pTRV2), and companion plasmids (pCVB and pTRV1) were cultured overnight in Luria–Bertani (LB) medium with 50 mg·L^−1^ rifampicin and 50 mg·L^−1^ kanamycin. After centrifugation (5000 rpm for 10 min), the precipitate was re-suspended in an infection buffer (10 mM MgCl_2_, 100 mM acetosyringone, and 10 mM MES, pH 5.6) to an OD_600_ of 0.8–1.0. Then, the infection buffers containing transformed *A. tumefaciens* were mixed in a 1:1 (v/v) ratio as follows: pCVA + pCVB, pCVA-*PsMIR159b* + pCVB, STTM159b + pCVB, TRV1 + TRV2, and TRV1+ TRV2-*PsMYB65*. They were left in the dark for approximately 3 h before infection.

For the transformation, sterilized buds were pre-cultured on 1/2 MS medium for 2 days prior to infection. Then, they were immersed in *A. tumefaciens* and subjected to vacuum infiltration at −0.9 kg cm^−2^ for 5 min, and then slowly deflated. After rinsing three times with sterile distilled water, the buds were then re-cultured on 1/2 MS medium, and maintained at 20°C (16/8 h light/dark). All primers used for the transient expression of flower buds are listed in Table S3 (see online supplementary material).

To determine the morphological changes in the transformed buds, images of buds were taken every 10 days using a digital camera (Canon EOS 400D; Japan). Bud height was measured using the ImageJ2 software (https://imagej.net/). Heights at 0, 10, and 20 DAI were recorded as H0, H1, and H2, respectively. Relative growth of the flower buds was calculated using the following formula: (H1 or H2 – H0)/H0 × 100%. At least three biological replicates (>10 buds per replicate) were used.

### RNA-seq analysis

Ten independent TRV2-*PsMYB65* and TRV2 control buds were evaluated by RT-qPCR, and three lines with silencing efficiency >50% were also selected for RNA-seq analysis. RNA concentration and integrity were determined using the Qubit 2.0 Fluorometer (Thermo Fisher Scientific, USA) and 2100 Bioanalyzer (Agilent Technologies, USA), and 1 μg of total RNA from each sample was used for cDNA library construction. Three biological replicates were performed using an Illumina HiSeq4000 platform. Approximately 6.0 Gb of clean data was generated per sample after low-quality read filtration (Table S2, see online supplementary material). For transcript assembly and annotation, Trinity 2.6.6 and Blast were used. DEGs were filtered based on | log_2_ (fold-change) | > 1 and adjusted *P* (q-value) <0.05, using DESeq2 1.22.2. KEGG and Gene Ontology analyses of DEGs were performed using the ‘ggplot’ package.

### Yeast assays

The transcriptional activation activity of PsMYB65 was assessed as previously described [55]. The CDS sequences of *PsMYB65*, *VP16*, *R2R3*, and *TRD* were cloned into the pGBKT7 vector and transformed into Y2H yeast strain. The pBD-VP16 and pBD empty vectors served as positive and negative controls, respectively. Transformants were screened on SD/−Trp/-His, SD/−Trp/-His/3-amino-1,2,4-triazole (3-AT), and SD/−Trp/-His/X-α-Gal.

For Y1H assay, CDS of *PsMYB65* was ligated into the pGADT7 vector (AD-PsMYB65), and the promoter fragments of *PsCYCD3;1* were inserted into the pHIS2 vector. These constructs were then transformed into Y187 yeast strain, and 3-AT was used to suppress the background expression of the pHIS2 vector. Transformants were selected on SD/−Trp, SD/−Trp/-His and SD/−Trp/-His/−Leu media. All primers used for yeast assays are listed in Table S3 (see online supplementary material).

### Dual-luciferase (LUC) assay

In order to determine the regulatory effects of PsMYB65 on its downstream candidate gene (*PsCYCD3;1*), the CDS of *PsMYB65* was cloned into the pGreenII0029 62-SK vector to construct the effector. The promoter fragments of *PsCYCD3;1* were inserted into the pGreenII 0800-LUC vector in order to generate reporters. These effectors and reporters were co-transfected into *N. benthamiana* leaves. The dual-LUC assay was performed as described previously [22]. All primers used are listed in Table S3 (see online supplementary material).

### Electrophoretic mobility shift assay (EMSA)

EMSA was performed as previously described by Zhang *et al.* [22]. Briefly, The CDS of *PsMYB65* was ligated into the pGEX-4 T-2 vector and then transformed into *Escherichia coli* strain BL21. The fusion protein was induced by adding isopropylthio-β-galactoside (0.5 mM) overnight at 16°C. The recombinant protein was then purified using a GST-Trap column (GE Healthcare, Pittsburgh, PA, USA). EMSA was performed using the LightShift Chemiluminescent EMSA Kit (Thermo Fisher Scientific), according to the manufacturer’s protocol. All probes used in EMSA are listed in Table S3 (see online supplementary material).

### Analysis of cell number and size

ImageJ was used to analyse cell number and size. Briefly, sections from TRV2-*PsMYB65*, TRV2 control, pCVA, and *OE-PsMIR159* transgenic buds after 20 DAI were imaged by Leica TCS-SP2 microscope. Then, three sections from each of three transgenic buds were used to calculate cell number and size.

### Statistical analyses

Student’s *t*-test was used to compare two groups, and one-way analysis of variance was used for multi-group comparisons. All statistical analyses were conducted using GraphPad Prism 7 (CA, USA).

## Acknowledgements

This work was supported by grants from National Natural Science Foundation of China (32271941, 31972452, 32371938), the Agricultural Seed Engineering Project of Shandong Province (2020LZGC011-1-4). The funding bodies had no role in the design of the study, the collection, analysis, and interpretation of data, or in writing the manuscript. We would like to thank Editage (www.editage.cn) for English language editing.

## Author contributions

TZ, XW, and YY: Writing–original draft, Validation, Methodology, Investigation, Formal analysis, Data curation. SZ and CL: Writing–original draft, Formal analysis, Investigation, Data curation. YZ: Writing–review & editing, Validation, Methodology, Conceptualization. SG: Writing–review & editing, Supervision, Project administration, Conceptualization.

## Data availability

The RNA-seq data have been deposited National Center for Biotechnology Information (NCBI) under accession number PRJNA813336. Sequence data used in this article can be found in the Arabidopsis Genome TAIR database or GenBank database under the following accession numbers: PsCYCD1;1 (OP142277), PsCYCD3;1 (OP142273), PsCYCD3;2 (OP142275), PsCYCD3;3 (OP142274), PsCYCD6;1 (OP142276), AtCYCD3 (AT4G34160), PtCYCD3;2 (CAN88857), AtCYCD3;3 (AT3G50070), AtCYCD3;2 (AT5G67260), PtCYCD6;1 (CAN88865), AtCYCD1;1 (AT1G70210), AtCYCD4;1 (AT5G65420), AtCYCD7;1 (AT5G02110), AtCYCD2;1 (AT2G22490), AtMYB97 (AT4G26930), AtMYB120 (AT5G55020), AtMYB101 (AT2G32460), PsMYB65 (MT211968), AtMYB65 (AT3G11440), AtMYB33 (AT5G06100), AtMYB104 (AT2G26950), AtMYB81 (AT2G26960).

## Conflict of interest statement

The authors declare no competing interests.

## Supplementary data

Supplementary data is available at *Horticulture Research* online.

## Supplementary Material

Web_Material_uhae052

## References

[1] Busov VB . Plant development: dual roles of poplar SVL in vegetative bud dormancy. Curr Biol. 2019;29:R68–70 30668953 10.1016/j.cub.2018.11.061

[2] Yang Q , Gao Y, Wu X. et al. Bud endodormancy in deciduous fruit trees: advances and prospects. Hortic Res. 2021;8:139 34078882 10.1038/s41438-021-00575-2 PMC8172858

[3] Maurya JP , Bhalerao RP. Photoperiod- and temperature-mediated control of growth cessation and dormancy in trees: a molecular perspective. Ann Bot. 2017;120:351–60 28605491 10.1093/aob/mcx061 PMC5591416

[4] Singh RK , Svystun T, AlDahmash B. et al. Photoperiod- and temperature-mediated control of phenology in trees – a molecular perspective. New Phytol. 2017;213:511–24 27901272 10.1111/nph.14346

[5] Böhlenius H , Huang T, Charbonnel-Campaa L. et al. CO/FT regulatory module controls timing of flowering and seasonal growth cessation in trees. Science. 2006;312:1040–3 16675663 10.1126/science.1126038

[6] Ruttink T , Arend M, Morreel K. et al. A molecular timetable for apical bud formation and dormancy induction in poplar. Plant Cell. 2007;19:2370–90 17693531 10.1105/tpc.107.052811 PMC2002631

[7] Tylewicz S , Tsuji H, Miskolczi P. et al. Dual role of tree florigen activation complex component FD in photoperiodic growth control and adaptive response pathways. Proc Natl Acad Sci USA. 2015;112:3140–5 25713384 10.1073/pnas.1423440112 PMC4364234

[8] Karlberg A , Bako L, Bhalerao RP. Short day-mediated cessation of growth requires the downregulation of AINTEGUMENTALIKE1 transcription factor in hybrid aspen. PLoS Genet. 2011;7:e1002361 22072988 10.1371/journal.pgen.1002361 PMC3207903

[9] Tylewicz S , Petterle A, Marttila S. et al. Photoperiodic control of seasonal growth is mediated by ABA acting on cell-cell communication. Science. 2018;360:212–5 29519919 10.1126/science.aan8576

[10] Zheng C , Halaly T, Acheampong AK. et al. Abscisic acid (ABA) regulates grape bud dormancy, and dormancy release stimuli may act through modification of ABA metabolism. J Exp Bot. 2015;66:1527–42 25560179 10.1093/jxb/eru519 PMC4339608

[11] Zhang T , Yuan Y, Zhan Y. et al. Metabolomics analysis reveals Embden Meyerhof Parnas pathway activation and flavonoids accumulation during dormancy transition in tree peony. BMC Plant Biol. 2020;20:1–17 33096979 10.1186/s12870-020-02692-x PMC7583197

[12] Singh RK , Miskolczi P, Maurya JP. et al. A tree ortholog of SHORT VEGETATIVE PHASE floral repressor mediates photoperiodic control of bud dormancy. Curr Biol. 2019;29:128–133.e2 30554900 10.1016/j.cub.2018.11.006

[13] Azeez A , Zhao YC, Singh RK. et al. EARLY BUD-BREAK 1 and EARLY BUD-BREAK 3 control resumption of poplar growth after winter dormancy. Nat Commun. 2021;12:1123 33602938 10.1038/s41467-021-21449-0 PMC7893051

[14] Singh RK , Maurya JP, Azeez A. et al. A genetic network mediating the control of bud break in hybrid aspen. Nat Commun. 2018;9:4173 30301891 10.1038/s41467-018-06696-y PMC6177393

[15] Zhang Y , Yuan Y, Liu Z. et al. GA_3_ is superior to GA_4_ in promoting bud endodormancy release in tree peony (*Paeonia suffruticosa*) and their potential working mechanism. BMC Plant Biol. 2021;65:323–32 10.1186/s12870-021-03106-2 PMC8256580 34225663

[16] Zheng C , Kwame Acheampong A, Shi Z. et al. Distinct gibberellin functions during and after grapevine bud dormancy release. J Exp Bot. 2018;69:1635–48 29385616 10.1093/jxb/ery022 PMC5888973

[17] Liu Q , Wu K, Harberd NP. et al. Green revolution DELLAs: from translational reinitiation to future sustainable agriculture. Mol Plant. 2021;14:547–9 33753307 10.1016/j.molp.2021.03.015

[18] Gao L , Niu D, Chi T. et al. PsRGL1 negatively regulates chilling- and gibberellin-induced dormancy release by PsF-box1-mediated targeting for proteolytic degradation in tree peony. Hortic Res. 2023;10:uhad044 10.1093/hr/uhad044 PMC10541556 37786434

[19] Ding Q , Zeng J, He XQ. Deep sequencing on a genome-wide scale reveals diverse stage-specific microRNAs in cambium during dormancy-release induced by chilling in poplar. BMC Plant Biol. 2014;14:267 25269469 10.1186/s12870-014-0267-6 PMC4189724

[20] Liu J , Guo X, Zhai T. et al. Genome-wide identification and characterization of microRNAs responding to ABA and GA in maize embryos during seed germination. Plant Biol. 2020;22:949–57 32526094 10.1111/plb.13142

[21] Zhang Y , Wang Y, Gao X. et al. Identification and characterization of microRNAs in tree peony during chilling induced dormancy release by high-throughput sequencing. Sci Rep. 2018;8:1–14 29540706 10.1038/s41598-018-22415-5 PMC5852092

[22] Zhang Y , Gao L, Wang Y. et al. Dual functions of PsmiR172b-PsTOE3 module in dormancy release and flowering in tree peony (*Paeonia suffruticosa*). Hortic Res. 2023;10:uhad033 10.1093/hr/uhad033 PMC10120838 37090095

[23] Yu J , Bennett D, Dardick C. et al. Genome-wide changes of regulatory non-coding RNAs reveal pollen development initiated at ecodormancy in peach. Front Mol Biosci. 2021;8:612881 33968979 10.3389/fmolb.2021.612881 PMC8098804

[24] Guo C , Xu Y, Shi M. et al. Repression of miR156 by miR159 regulates the timing of the juvenile-to-adult transition in Arabidopsis. Plant Cell. 2017;29:1293–304 28536099 10.1105/tpc.16.00975 PMC5502449

[25] Gocal GFW , Sheldon CC, Gubler F. et al. GAMYB-like genes, flowering, and gibberellin signaling in Arabidopsis. Plant Physiol. 2001;127:1682–93 11743113 PMC133573

[26] Millar AA , Gubler F. The Arabidopsis GAMYB-like genes, MYB33 and MYB65, are microRNA-regulated genes that redundantly facilitate anther development. Plant Cell. 2005;17:705–21 15722475 10.1105/tpc.104.027920 PMC1069693

[27] Ko SS , Li MJ, Ho YC. et al. Rice transcription factor GAMYB modulates bHLH142 and is homeostatically regulated by TDR during anther tapetal and pollen development. J Exp Bot. 2021;72:4888–903 33940615 10.1093/jxb/erab190

[28] Gai S , Zhang Y, Liu C. et al. Transcript profiling of *Paoenia ostii* during artificial chilling induced dormancy release identifies activation of GA pathway and carbohydrate metabolism. PLoS One. 2013;8:e55297 23405132 10.1371/journal.pone.0055297 PMC3566188

[29] Huang X , Zhu W, Dai S. et al. The involvement of mitochondrial phosphate transporter in accelerating bud dormancy release during chilling treatment of tree peony (*Paeonia suffruticosa*). Planta. 2008;228:545–52 18566830 10.1007/s00425-008-0757-6

[30] Yan J , Gu Y, Jia X. et al. Effective small RNA destruction by the expression of a short tandem target mimic in Arabidopsis. Plant Cell. 2012;24:415–27 22345490 10.1105/tpc.111.094144 PMC3315224

[31] Wu HJ , Ma YK, Chen T. et al. PsRobot: a web-based plant small RNA meta-analysis toolbox. Nucleic Acids Res. 2012;40:W22–8 22693224 10.1093/nar/gks554 PMC3394341

[32] Lescot M , Déhais P, Thijs G. et al. PlantCARE, a database of plant cis-acting regulatory elements and a portal to tools for in silico analysis of promoter sequences. Nucleic Acids Res. 2002;30:325–7 11752327 10.1093/nar/30.1.325 PMC99092

[33] Achard P , Herr A, Baulcombe DC. et al. Modulation of floral development by a gibberellin-regulated microRNA. Development. 2004;131:3357–65 15226253 10.1242/dev.01206

[34] da Silva EM , Silva GFF, Bidoia DB. et al. microRNA159-targeted SlGAMYB transcription factors are required for fruit set in tomato. Plant J. 2017;92:95–109 28715118 10.1111/tpj.13637

[35] Gao J , Chen H, Yang H. et al. A brassinosteroid responsive miRNA-target module regulates gibberellin biosynthesis and plant development. New Phytol. 2018;220:488–501 30009574 10.1111/nph.15331

[36] Zheng Z , Wang N, Jalajakumari M. et al. MiR159 represses a constitutive pathogen defense response in tobacco. Plant Physiol. 2020;182:2182–98 32041907 10.1104/pp.19.00786 PMC7140937

[37] Mathiason K , He D, Grimplet J. et al. Transcript profiling in *Vitis riparia* during chilling requirement fulfillment reveals coordination of gene expression patterns with optimized bud break. Funct Integr Genomics. 2009;9:81–96 18633655 10.1007/s10142-008-0090-y

[38] Dong QK , Hu BB, Zhang C. microRNAs and their roles in plant development. Front Plant Sci. 2022;13:824240 35251094 10.3389/fpls.2022.824240 PMC8895298

[39] Azeez A , Miskolczi P, Tylewicz S. et al. A tree ortholog of APETALA1 mediates photoperiodic control of seasonal growth. Curr Biol. 2014;24:717–24 24656832 10.1016/j.cub.2014.02.037

[40] Hu Z , Shen X, Xiang X. et al. Evolution of MIR159/319 genes in *Brassica campestris* and their function in pollen development. Plant Mol Biol. 2019;101:537–50 31745746 10.1007/s11103-019-00920-z

[41] Wang C , Jogaiah S, Zhang W. et al. Spatio-temporal expression of miRNA159 family members and their GAMYB target gene during the modulation of gibberellin-induced grapevine parthenocarpy. J Exp Bot. 2018;69:3639–50 29905866 10.1093/jxb/ery172

[42] Jing WK , Gong FF, Liu GQ. et al. Petal size is controlled by the MYB73/TPL/HDA19-miR159-CKX6 module regulating cytokinin catabolism in *Rosa hybrida*. Nat Commun. 2023;14:7106 37925502 10.1038/s41467-023-42914-y PMC10625627

[43] Wojtania A , Markiewicz M, Waligórski P. Regulation of the bud dormancy development and release in micropropagated rhubarb 'Malinowy'. Int J Mol Sci. 2022;23:1480 35163404 10.3390/ijms23031480 PMC8835828

[44] Shimotohno A , Aki SS, Takahashi N. et al. Regulation of the plant cell cycle in response to hormones and the environment. Annu Rev Plant Biol. 2021;72:273–96 33689401 10.1146/annurev-arplant-080720-103739

[45] Vandepoele K , Raes J, De Veylder L. et al. Genome-wide analysis of core cell cycle genes in Arabidopsis. Plant Cell. 2002;14:903–16 11971144 10.1105/tpc.010445 PMC150691

[46] Achard P , Gusti A, Cheminant S. et al. Gibberellin signaling controls cell proliferation rate in Arabidopsis. Curr Biol. 2009;19:1188–93 19576768 10.1016/j.cub.2009.05.059

[47] Xue T , Liu Z, Dai X. et al. Primary root growth in *Arabidopsis thaliana* is inhibited by the miR159 mediated repression of MYB33, MYB65 and MYB101. Plant Sci. 2017;262:182–9 28716415 10.1016/j.plantsci.2017.06.008

[48] Guan C , Xue Y, Jiang P. et al. Overexpression of ptocycd3;3 promotes growth and causes leaf wrinkle and branch appearance in *Populus*. Int J Mol Sci. 2021;22:1–21 10.3390/ijms22031288 PMC7866192 33525476

[49] Zhang Y , Yu D, Liu C. et al. Dynamic of carbohydrate metabolism and the related genes highlights PPP pathway activation during chilling induced bud dormancy release in tree peony (*Paeonia suffruticosa*). Sci Hortic (Amsterdam). 2018;242:36–43

[50] Kumar S , Stecher G, Tamura K. MEGA7: molecular evolutionary genetics analysis version 7.0 for bigger datasets. Mol Biol Evol. 2016;33:1870–4 27004904 10.1093/molbev/msw054 PMC8210823

[51] Chen C , Chen H, Zhang Y. et al. TBtools: an integrative toolkit developed for interactive analyses of big biological data. Mol Plant. 2020;13:1194–202 32585190 10.1016/j.molp.2020.06.009

[52] Liu W , Xie Y, Ma J. et al. IBS: an illustrator for the presentation and visualization of biological sequences. Bioinformatics. 2015;31:3359–61 26069263 10.1093/bioinformatics/btv362 PMC4595897

[53] Livak KJ , Schmittgen TD. Analysis of relative gene expression data using real-time quantitative PCR and the 2^-ΔΔCT^ method. Methods. 2001;25:402–8 11846609 10.1006/meth.2001.1262

[54] Jefferson RA , Kavanagh TA, Bevan MW. GUS fusions: beta-glucuronidase as a sensitive and versatile gene fusion marker in higher plants. EMBO J. 1987;6:3901–7 3327686 10.1002/j.1460-2075.1987.tb02730.x PMC553867

[55] Chen J , Li Y, Li Y. et al. Auxin response factor 18–histone deacetylase 6 module regulates floral organ identity in rose (*Rosa hybrida*). Plant Physiol. 2021;186:1074–87 33729501 10.1093/plphys/kiab130 PMC8195501

