# Supplementary material for: PsmiR159b-PsMYB65 module functions in the resumption of bud growth after endodormancy by affecting the cell cycle in tree peony
Source: Hortic Res. 2024 Feb 23;11(4):uhae052. doi: 10.1093/hr/uhae052 (PMC11025381; doi:10.1093/hr/uhae052)
Supplement: Web_Material_uhae052 [file web_material_uhae052.zip › supporting figures-24.1.6.docx]

**PsmiR159b-*PsMYB65* module functions in the resumption of budbreak bud growth after endodormancy by affecting cell cycle in tree peony**

Tao Zhang^#1,2,3^, Xinyu Wang^#1,2^, Yanchao Yuan^1,2^, Shoujie Zhu^1,2^, Chunying Liu^1,2^, Yuxi Zhang^*1,2^, Shupeng Gai^*1,2^

**Supplemental Material**

Supplemental Figure S1. Heatmap of PsmiR159a, b, and c expression during chilling-induced dormancy release by Illumina sequencing.

Supplemental Figure S2. Structure of STTM159b.

Supplemental Figure S3. Genome-wide identification of *PsCYCD* family.

Supplemental Figure S4. Phylogenetic analysis of the Arabidopsis MYB family and other plant GAMYB proteins.

Supplemental Figure S5. Identification and expression analysis of PsMYB65.

Supplemental Figure S6. The expression pattern of *PsCYCD3;1* after GA_3_ treatment.

Supplemental Figure S7. Expression patterns of miR159s in floral organ of *Arabidopsis thaliana* .


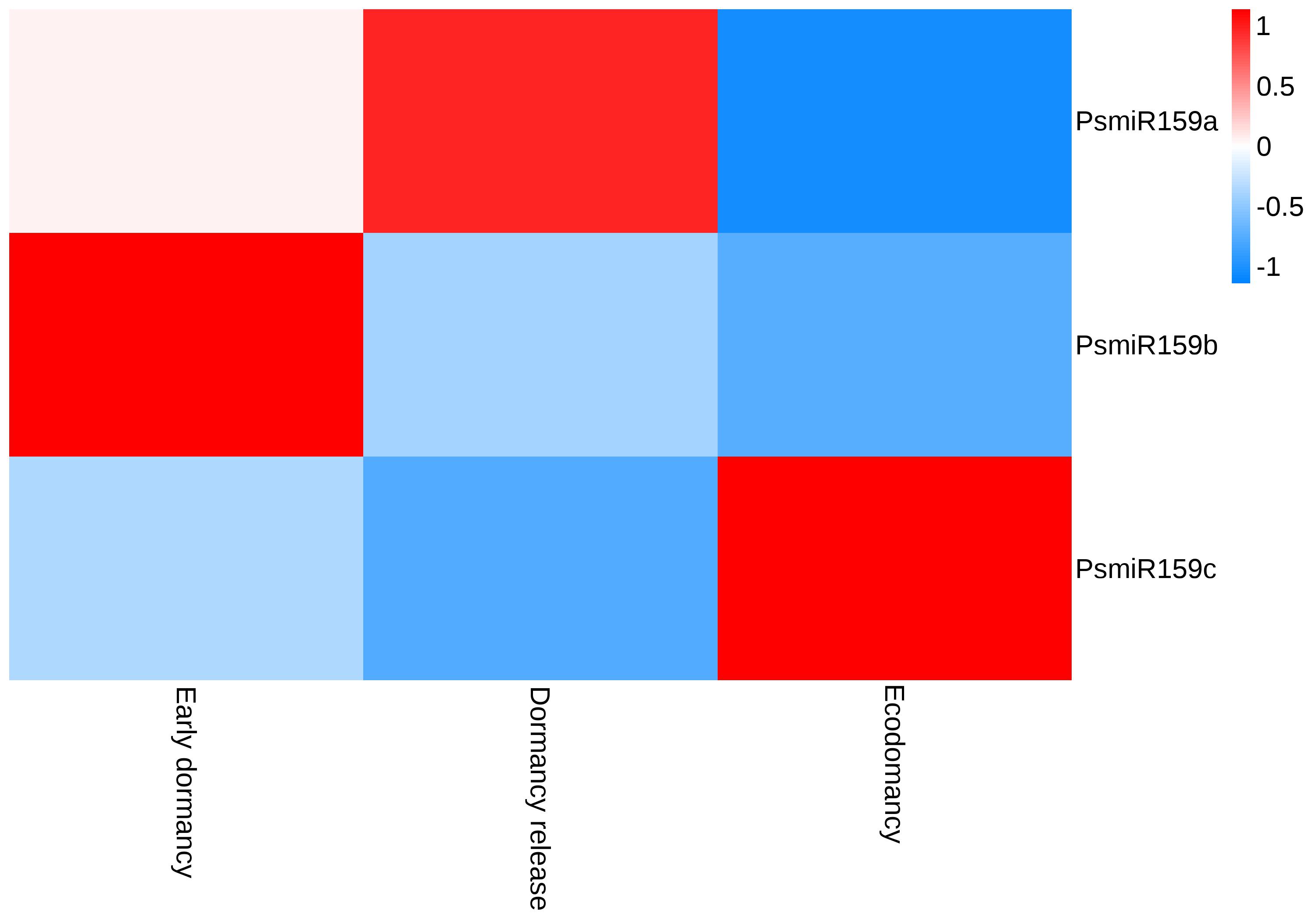


Supplemental Figure S1. Heatmap of PsmiR159a, b, and c expression during chilling-induced dormancy release by Illumina sequencing. PsmiR159a, b and c were identified in the tree peony genome. Illumina sequenced data were aligned to the three microRNAs, and related read numbers were recorded and normalized as their expression abundance. Heatmap was constructed according to their abundance. Notably, sequences of putative introns and exons were discarded in advance.


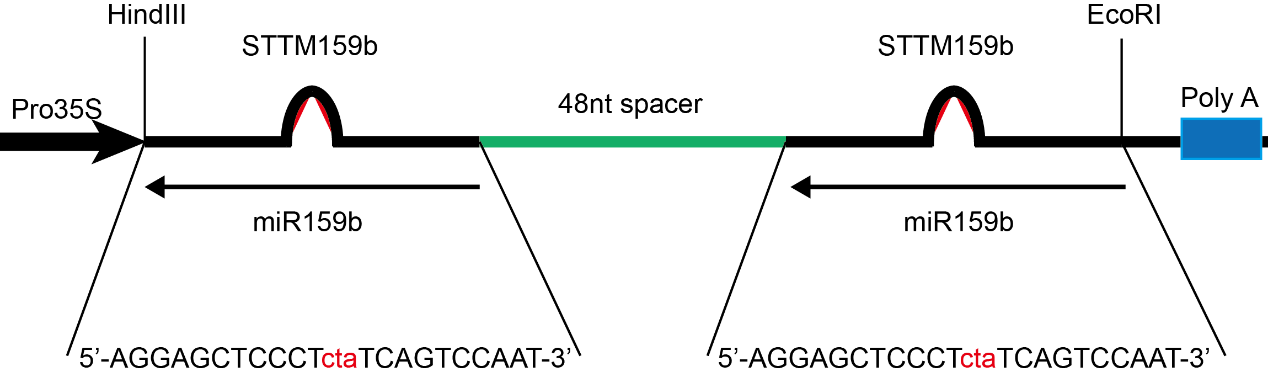


Supplemental Figure S2. Structure of STTM159b. The insert ‘cta’ bases were used to avoided cleavage by miR159.


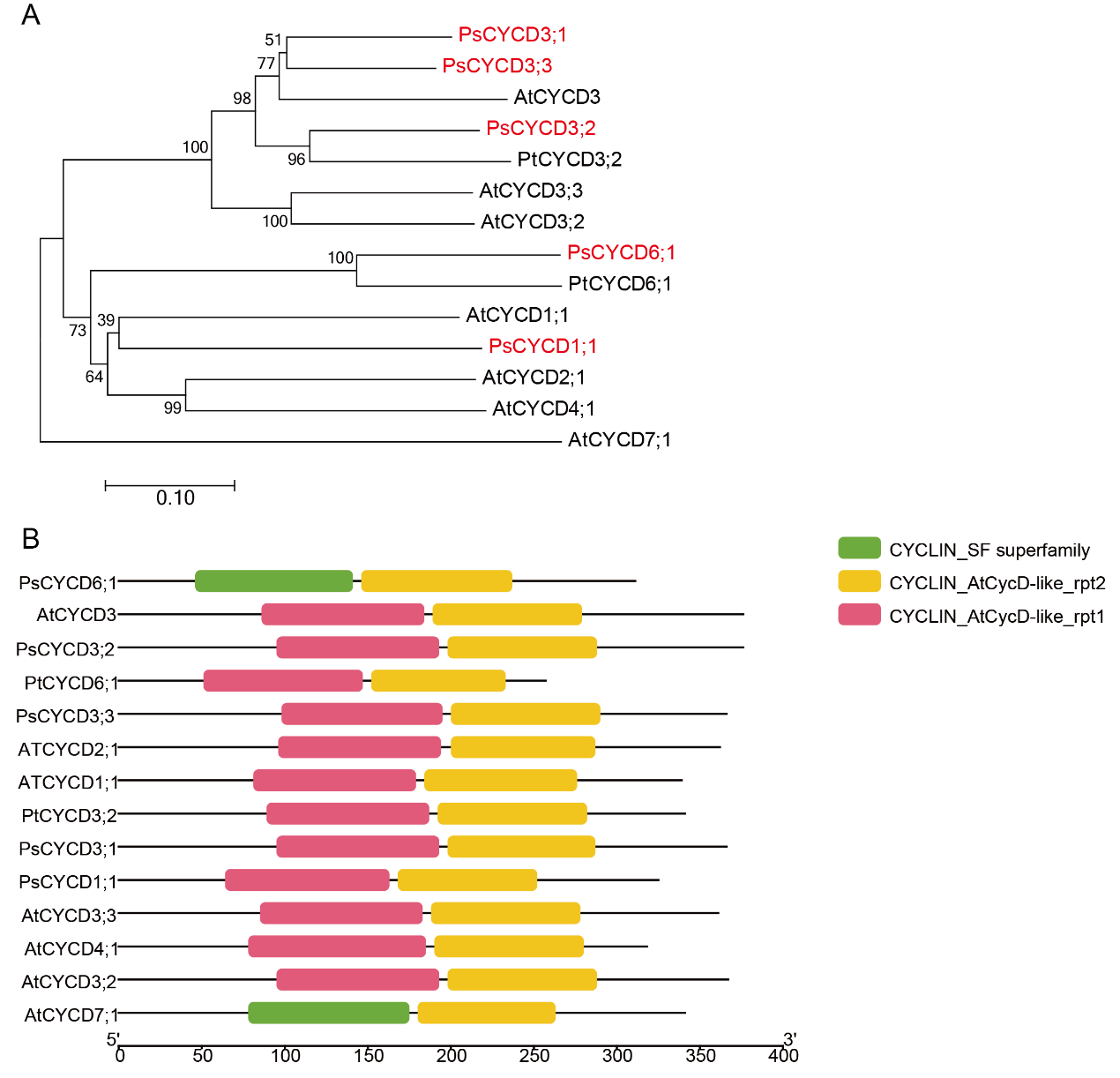


Supplemental Figure S3. Genome-wide identification of *PsCYCD* family. A, Phylogenetic analysis of CYCD homologs in tree peony and *Arabidopsis thaliana*. CYCD homologs in tree peony were marked with red font. B, Conservative domains of CYCD homologs in tree peony and *A. thaliana*.


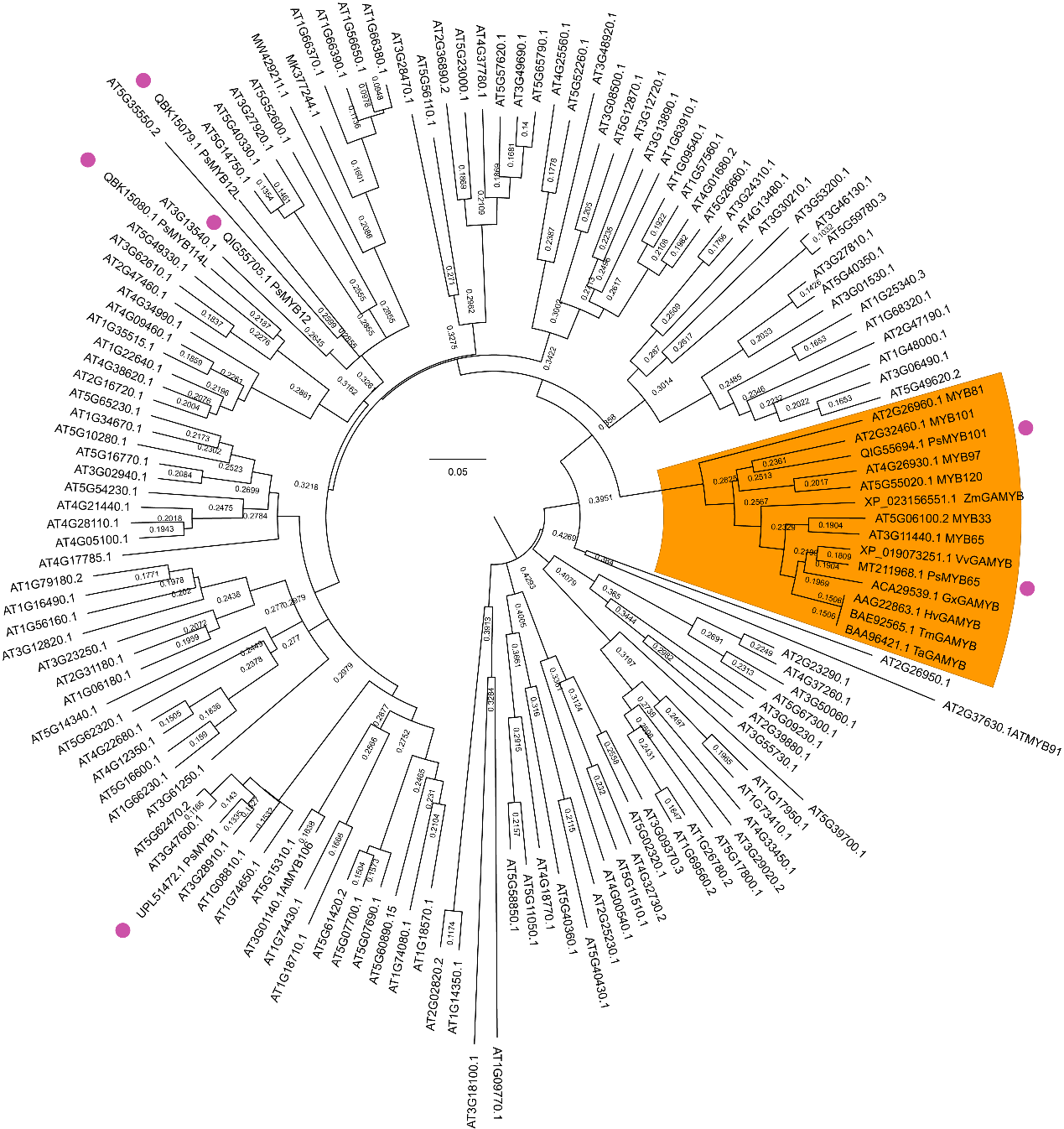


Supplemental Figure S4. Phylogenetic analysis of the Arabidopsis MYB family and other plant GAMYB proteins. Red spots indicate tree peony MYB proteins and orange shading indicates members of the GAMYB family.


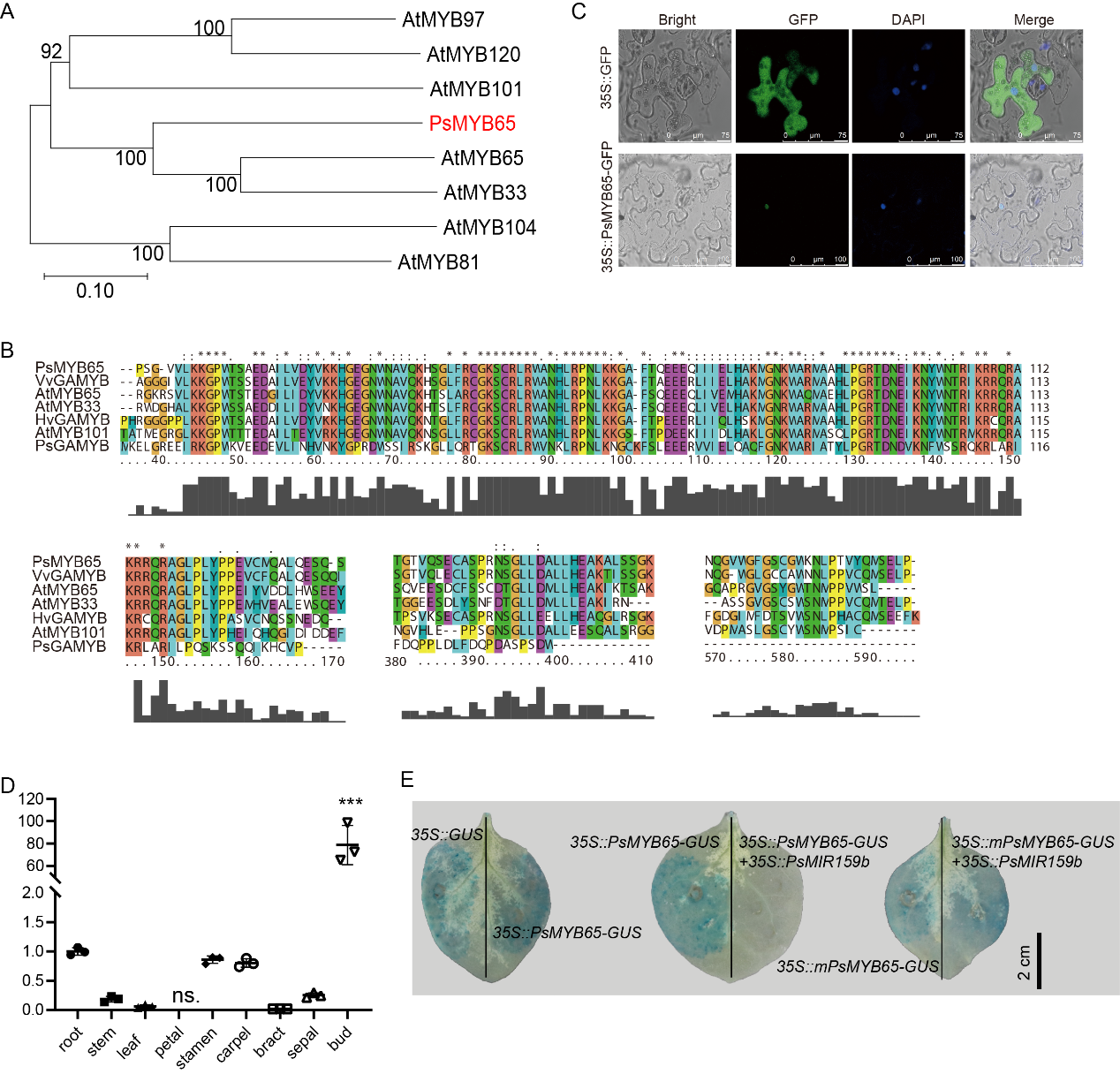


Supplemental Figure S5. Identification and expression analysis of PsMYB65. A, Phylogenetic analysis of PsMYB65 with the other MYB proteins in *Arabidopsis*. Red marked PsMYB65 proteins of tree peony. B. Amino acid alignment of PsMYB65 homologs in different species. C, Subcellular localization of PsMYB65. Nucleus are indicated by DAPI. D, The expression levels of *PsMYB65* in different tissues. The tissues were from tree peony plantlets at the early stage of flowering except the buds. The buds were mixed with 0, 7, 14 and 21 DAC. Data were shown as mean ± SD (n = 3) from three biological replicates (five buds in each replicate). Asterisk (*) indicated statistically significant differences via one-way ANOVA (***P < 0.001). E, Histochemical staining of tobacco leaves transformed with *35S::GUS*, *35S::PsMYB65-GU*S, *35S::mPsMYB65-GUS*, *35S::PsMYB65-GUS +35S::PsMIR159b*, and *35S::mPsMYB65-GUS*+ *35S:: PsMIR159b*. Scale bar, 2 cm.


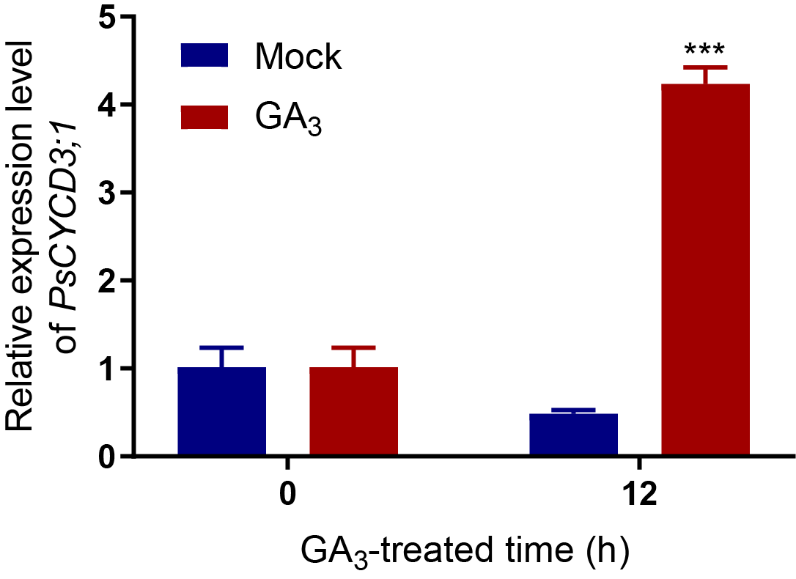


Supplemental Figure S6. The expression pattern of *PsCYCD3;1* after GA_3_ treatment. The dormant buds (7 DAC) were treated by GA_3_. Data were shown as mean ± SD (n > 3) and asterisks indicate significant differences by two-tailed Student’s t-test (****P* < 0.001).


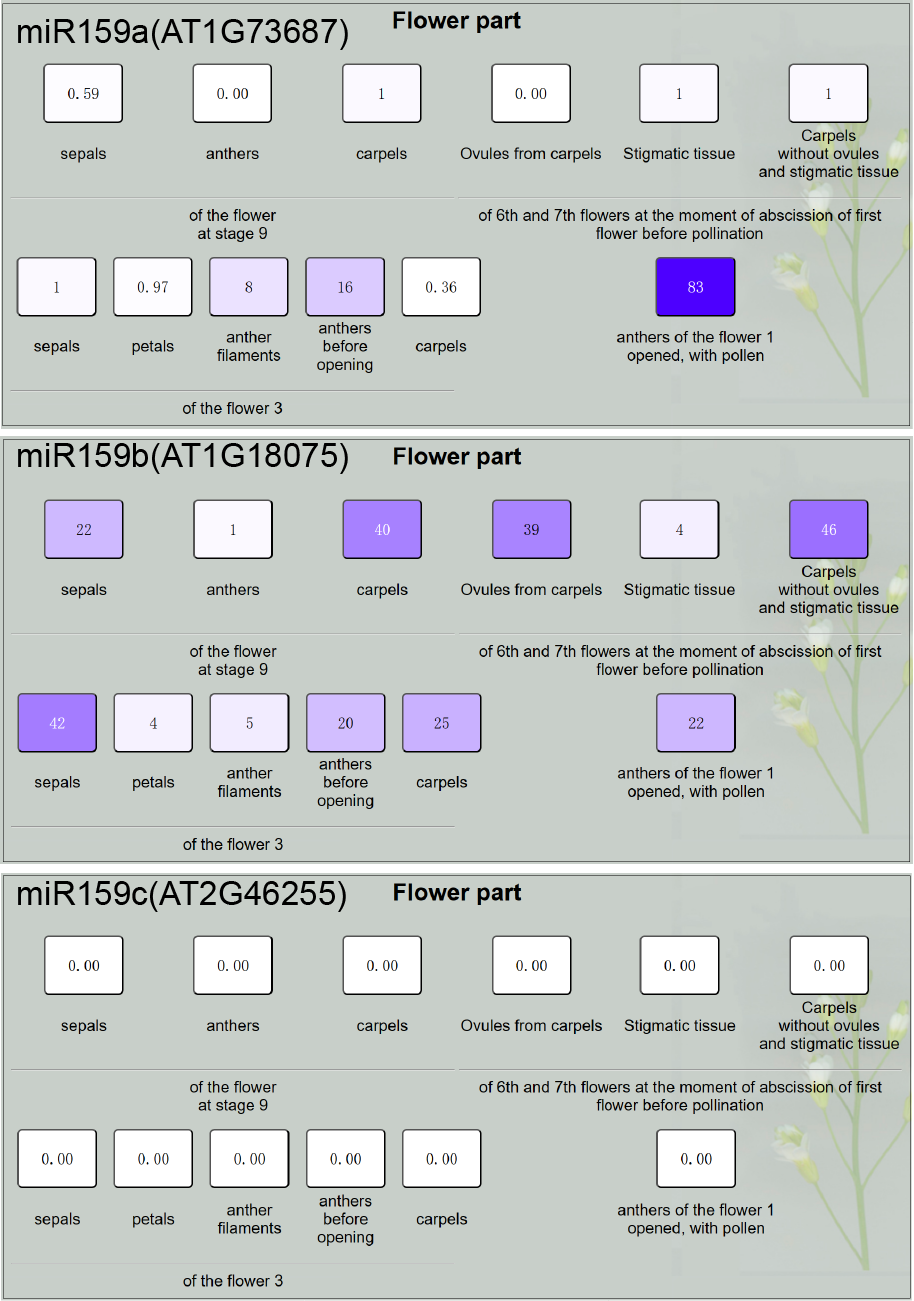


Supplemental Figure S7. Expression patterns of miR159s in floral organs of *Arabidopsis thaliana*. Data were sourced from TAIR (https://www.arabidopsis.org/).
