# Supplementary material for: PsmiR159b-PsMYB65 module functions in the resumption of bud growth after endodormancy by affecting the cell cycle in tree peony
Source: Hortic Res. 2024 Feb 23;11(4):uhae052. doi: 10.1093/hr/uhae052 (PMC11025381; doi:10.1093/hr/uhae052)
Supplement: Web_Material_uhae052 [file web_material_uhae052.zip › Supporting file 1-24.1.11.docx]

PsmiR159b-*PsMYB65* module regulates bud endodormancy release by affecting cell cycle in tree peony

**Supplemental Material**

Supplemental file S1. Precursor sequences of *PsMIR159s*.

Supplement File S1. The precursor sequences of *PsMIR159s*. The underline indicated the mature miRNA sequence.

>pre-miR159a

TGGAGTGGAGCTCCTTGAAGTCCAATAAAGGGTGTTACTGAGTGGATTGAGCTGCTGAATTATGGATCCCAGAGCCCTACCCTTGATGAAATCATATGGGTAGGCTTGTGGCTTGCATAACTCAGGAGCTGCATTACTCGAGTTAGATCCTTGTTTGGATTGAAGGGAGCTCTACATCAC

>pre-miR159b

TTGGGAGCAGAGGAGCTCCTTTCATTCCAATACGAAGGGCTGGAATGCGGGCAGAGCTGCCATCTCATGCATAAGGCTATGCTTATTATTCTTTATGATCAGAGTTGGAGAGAATGAATGGATTTGCGAAATTAAGCTTAGCCTGGTGCATGGTGTGGGAGCAACTCCTTCCGCATCTTTACCCTACATATTGGAGTGAAGGGAGCTCCTGG

>pre-miR159c

GGAAGTGAGGAGCTCCTTTTGGTCCAATACCGAGGGCTGAGATGCGGATAGAGCTGCCATCTCATGCATAAAGCTATGCTTAACCCTTGTGTAGTGATGTTAAGCTTAGCCATTGCATGATGTGGGAGCAACTCGTTCTGTATACTTCGTCCCCAAATTGGACTGAAGGGAGCTCCTCATGA
