# Supplementary material for: PsmiR159b-PsMYB65 module functions in the resumption of bud growth after endodormancy by affecting the cell cycle in tree peony
Source: Hortic Res. 2024 Feb 23;11(4):uhae052. doi: 10.1093/hr/uhae052 (PMC11025381; doi:10.1093/hr/uhae052)
Supplement: Web_Material_uhae052 [file web_material_uhae052.zip › SupportingTables-20240110.docx]

PsmiR159b-*PsMYB65* module regulates bud endodormancy release by affecting cell cycle in tree peony

**Supplemental Material**

Supplemental Table S1. Predicted target genes of PsmiR159b in tree peony.

Supplemental Table S2. RNA-seq between TRV2-PsMYB65 buds and TRV2 control.

Supplemental Table S3. List of primers used in this study.

Supplement Table S1. The predicted target genes of PsmiR159b in tree peony.

| miRNA id | Target id | Expectation | Target  start | Target  end | Target aligned fragment | Inhibition | Target Describetion |
| --- | --- | --- | --- | --- | --- | --- | --- |
| psu-miR159b | HQ_transcript_11644 | 2.5 | 516 | 536 | UGAGGCUUCCUUCGAUCCAAU | Cleavage | Hypothetical protein |
| psu-miR159b | HQ_transcript_14546 | 3 | 1255 | 1275 | UGGAGCUCCAUUCGAUCCAAA | Cleavage | MYB transcription factor |
| psu-miR159b | HQ_transcript_24507 | 3 | 1200 | 1220 | AGGGGGACCCUUCAGUCCAAU | Cleavage | Transcription factor TCP2 |
| psu-miR159b | HQ_transcript_37127 | 3 | 42 | 62 | CGGGUCUCUCUUUAAUCCGAU | Cleavage | Hypothetical protein ES319_A02G100300v1 |
| psu-miR159b | HQ_transcript_24139 | 3 | 280 | 300 | UCGAGCUCUUUUCAAUUGAAU | Cleavage | formin-like protein 1 |
| psu-miR159b | HQ_transcript_6257 | 3 | 1441 | 1461 | UCGAGCUCUUUUCAAUUGAAU | Cleavage | Hypothetical protein F0562_034672 |

Supplement Table S2. RNA-seq between TRV2-PsMYB65 buds and TRV2 control

| **Sample** | **Raw Reads** | **Clean Reads** | **Clean Base(G)** | **Error Rate (%)** | **Q20(%)** | **GC Content (%)** |
| --- | --- | --- | --- | --- | --- | --- |
| TRV2-1 | 48591504 | 47150010 | 7.07 | 0.03 | 96.05 | 44.85 |
| TRV2-2 | 45491120 | 44473396 | 6.67 | 0.03 | 96.01 | 44.65 |
| TRV2-3 | 43369870 | 42151904 | 6.32 | 0.03 | 96.07 | 44.61 |
| TRV2-MYB65-1 | 44088782 | 42842764 | 6.43 | 0.03 | 95.94 | 44.87 |
| TRV2-MYB65-2 | 46282050 | 45072904 | 6.76 | 0.03 | 96.55 | 45.23 |
| TRV2-MYB65-3 | 46964812 | 45424720 | 6.81 | 0.03 | 96.26 | 44.71 |

Supplement Table S3. List of the primers used in this study.

| Oligo names | Sequences (5'-3') | Purposes |
| --- | --- | --- |
| PsMYB65-qRT-F | AACACCCGAATCAAGAGACG | qRT-PCR |
| qqPsMYB65-qRT-R | CCGAGTCCTTTCATCAGCAT |  |
| PsCYCD3;1-qRT-F | AGCAATGGGCACAGCAAAAG |  |
| PsCYCD3;1-qRT-R | CACATCCACCAGAAACCCGA |  |
| pre-miR159a-qRT-F | TGGAGTGGAGCTCCTTGAAGTCC |  |
| pre-miR159a-qRT-R | GTGATGTAGAGCTCCCTTCAATCC |  |
| pre-miR159b-qRT-F | GGAAGTGAGGAGCTCCTTTTGGTC |  |
| pre-miR159b-qRT-R | TCATGAGGAGCTCCCTTCAGTCC |  |
| pre-miR159c-qRT-F | TTGGGAGCAGAGGAGCTCCTTTC |  |
| pre-miR159c-qRT-R | CCAGGAGCTCCCTTCACTCCAATATG |  |
| miR159a-qRT-F | CGCGTTTGGATTGAAGGGA |  |
| miR159a-qRT-R | GTCGTATCCAGTGCAGGGTCCGAGGTATTCGCACTGGATACGACTAGAGC |  |
| miR159b-qRT-F | CGCGATTGGACTGAAGGGA |  |
| miR159b-qRT-R | GTCGTATCCAGTGCAGGGTCCGAGGTATTCGCACTGGATACGACAGGAGC |  |
| miR159c-qRT-F | CGCGATTGGAGTGAAGGGA |  |
| miR159c-qRT-R | GTCGTATCCAGTGCAGGGTCCGAGGTATTCGCACTGGATACGACAGGAGC |  |
| TRV2-MYB65-F | aaggttaccgaattctctagaGCAGTGGGAAGAATAACTATTCGTC | VIGS |
| TRV2-MYB65-R | gagacgcgtgagctcggtaccTCGTAGGCTTGTAGTTGTTACCATAA |  |
| TRV2-CYCD3;1-F | aaggttaccgaattctctagaTAATGTAAGCTCAGATGGGCCTT |  |
| TRV2-CYCD3;1-F | gagacgcgtgagctcggtaccTCCATTCTCTGAATAGTCTTTGCTTC |  |
| BD-MYB65-R2R3-F | tcagaggaggacctgcatatgATGAGTCGATTAACTAATGATAGCGAC | Yeast assays |
| BD-MYB65-R2R3-R | tcgacggatccccgggaattcACGTTGACGTCTCTTGATTCGG |  |
| BD-MYB65-TRD-F | tcagaggaggacctgcatatgGCTGGCTTGCCTCTCTATCCT |  |
| BD-MYB65-TRD-R | tcgacggatccccgggaattcAGGTAGTTCAGACATTTGATACACAGTAGG |  |
| AD-MYB65-F | gccatggaggccagtgaattcATGAGTCGATTAACTAATGATAGCGAC |  |
| AD-MYB65-R | cagctcgagctcgatggatccAGGTAGTTCAGACATTTGATACACAGTAGG |  |
| pHis-proCYCD3;1-P1-F | gactcactatagggcgaattcCGACCCCATTTGTGTGGGA |  |
| pHis-proCYCD3;1-P1-R | attactagtggatccacgcgtCGAAATAATTATACTTCCAAAAATACCC |  |
| pHis-proCYCD3;1-P2-F | gactcactatagggcgaattcCGAAATTTTTGGCCAATTTTG |  |
| pHis-proCYCD3;1-P2-R | attactagtggatccacgcgtCAAACACACCACATACTGCTGCC |  |
| luc-proCYCD3;1-F | gtcgacggtatcgataagcttCGAGGGCAAATGGAAGATGA | Dual-LUC |
| luc-proCYCD3;1-R | agaactagtggatcccccgggCTTCTTCTGAGAAGAAGAGACCAAAGC |  |
| PsMYB65-F | ATGAGTCGATTAACTAATGATAGCGACGATGG | Mutation of PsMYB65 |
| PsMYB65-Rm | TAGACTTGGTAGTTCTAATTTCTCGGCACCGGGCTTTGAATT |  |
| PsMYB65-Fm | TAGAACTACCAAGTCTACAATTTCTGGAGCCAGATTTAGGTAGC |  |
| PsMYB65-R | TCAAGGTAGTTCAGACATTTGATACACAGT |  |
| PsCYCD3;1-Cold-F | ATTTTTGGCCAATTTTGGTTAAATTTCAAAATTTGT | EMSA |
| PsCYCD3;1-Cold-R | ACAAATTTTGAAATTTAACCAAAATTGGCCAAAAAT |  |
| PsCYCD3;1-mutant-F | ATTTTTGGCCAATAAAAAAAAAATTTCAAAATTTGT |  |
| PsCYCD3;1-mutant-R | ACAAATTTTGAAATTTTTTTTTTATTGGCCAAAAAT |  |
| PsCYCD3;1-Bio-F | Biotin-ATTTTTGGCCAATTTTGGTTAAATTTCAAAATTTGT |  |
| PsCYCD3;1-Bio-R | Biotin-ACAAATTTTGAAATTTAACCAAAATTGGCCAAAAAT |  |
| GST-MYB65-F | cgcgtggatccccaggaattcATGAGTCGATTAACTAATGATAGCGAC |  |
| GST-MYB65-R | gtcacgatgcggccgctcgagAGGTAGTTCAGACATTTGATACACAGTAGG |  |
| pSuper1300-PsMYB65-F | atacaccaaatcgactctagaATGAGTCGATTAACTAATGATAGCGAC | Subcellular  localization of PsMYB65 |
| pSuper1300-PsMYB65-R | gcccttgctcaccatggtaccAGGTAGTTCAGACATTTGATACACAGTAGG |  |
